# Fibrinogen‐Like Protein 2 Modulates B Cell Mucosal Immunity by Suppressing Receptor for Activated C‐Kinase 1‐Mediated AKT Phosphorylation

**DOI:** 10.1002/mco2.70633

**Published:** 2026-03-24

**Authors:** Jiang Chang, Da Huang, Wei Yuan, Jianing Tang, Jingzhi Yang, Yuying Chen, Zhize Yuan, Yizhi Wu, Di Wu, Weiming Yan, Qin Ning

**Affiliations:** ^1^ Department and Institute of Infectious Disease Tongji Hospital, Tongji Medical College and State Key Laboratory for Diagnosis and Treatment of Severe Zoonotic Infectious Diseases, Huazhong University of Science and Technology Wuhan Hubei Province PR China; ^2^ Department of Orthopedics Qilu Hospital of Shandong University Jinan Shandong PR China

**Keywords:** B cell, Fgl2, IgA, mucosal immunity, Rack1

## Abstract

Fibrinogen‐like protein 2 (Fgl2) is a critical immunoregulatory factor, yet its precise roles in B‐cell biology and mucosal immunity remain largely undefined. In this study, utilizing *Fgl2*‐knockout (KO) mice, we identified novel B cell subsets in the spleen (SPL), predominantly characterized by IGHA clonal dominance. Employing an intestinal *Trichinella spiralis* (*T. s*) infection model and samples from patients exhibiting mucosal immune responses (the early stage of COVID‐19 infection), we investigated the function of Fgl2 in mucosal immunity. We demonstrate that Fgl2 directly interacts with Receptor for activated C‐kinase 1 (Rack1), thereby attenuating B cell receptor (BCR) signaling and metabolic activity by inhibiting AKT phosphorylation. Furthermore, the Fgl2 deficiency‐induced expansion of marginal zone (MZ) B cells, germinal center (GC) B cells, and IgA+ plasma cells was effectively counteracted by in vivo Rack1 inhibition. Consistently, a Rack1 inhibitor also abrogated the enhanced activation of Fgl2‐deficient B cells in vitro. Fgl2 deficiency also augmented early B cell activation, including B cell spreading, clustering, and signalosome recruitment, through upregulation of the DOCK8‐WASP‐actin axis. Our research uncovers an intrinsic role for Fgl2 in regulating BCR signaling, B cell differentiation, and mucosal immunity, elucidating a key underlying molecular mechanism.

## Graphical abstract

In our study, B cells are activated with the assistance of Breg and Tfh cells, subsequently differentiating into IgA+ plasma cells. These plasma cells produce secretory IgA (sIgA) at mucosal surfaces to combat viral and parasitic infections. Mechanistically, Fgl2 directly binds to Rack1. This interaction influences B‐cell metabolism by inhibiting AKT phosphorylation. Concurrently, it also impacts B‐cell activation by attenuating B‐cell receptor (BCR) signaling, including critical early events such as B‐cell spreading, clustering, and signalosome recruitment.

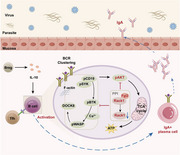

## 1 Introduction

Fibrinogen‐like protein‐2 (Fgl2) is a 64–70 kD type 2 transmembrane protein with 439 amino acids (AAs) [1]. The N‐terminus contains a 2 AA‐long cytoplasmic domain and a 21‐AA‐long transmembrane domain, with the remaining 416 AAs constituting the extracellular domain [2]. Known as an immunomodulator, Fgl2 plays crucial roles in innate and adaptive immunity, and its expression is induced by viral proteins or cytokines during infections and serves as an immunosuppressive molecule. Fgl2 was identified as a secreted protein from T follicular regulatory cells; it directly binds to Fc γ receptors (FcγR) IIB on B cells and limits B cell survival and class‐switch recombination. Fgl2 deficiency leads to dysregulated antibody responses [3]. This indicates that Fgl2 signaling displays a crucial role in B‐cell development, but limited knowledge is available on the intrinsic role of Fgl2 in B cells. Accordingly, targeted deletion of Fgl2 in mice led to an augmented population of antibody‐producing B cells and DCs within the spleen (SPL). Furthermore, the antiviral B cell response postinfection with lymphocytic choriomeningitis virus was significantly amplified in Fgl2^−/−^ mice, positively contributing to the clearance of the virus. The diverse functions of Fgl2, particularly in the context of immune regulation, have been extensively studied [4]; however, its role in B‐cell activation, development, and mucosal immunity remains relatively unexplored.

Receptor for activated C‐kinase 1 (Rack1), a member of the WD40 protein family, is recognized for its role as a scaffolding protein within complex interaction networks [5]. Consequently, Rack1 has been identified to interact with a multitude of partners, encompassing kinases, signaling proteins, membrane‐bound receptors, and ion channels. Additionally, Rack1 has been characterized as a ubiquitous ribosomal protein found in the ribosomes of all eukaryotic organisms [6]. Moreover, Rack1 has been found to play crucial roles in B‐cell homeostasis and BCR activation. Mb1‐driven Rack1 deficiency almost completely blocks B‐cell development at the pro‐B‐cell stage [7].

Mucosal immunity is a critical component of the host defense system, providing a frontline barrier against the vast array of pathogens that inhabit our body's mucosal surfaces [8]. Understanding the complex interactions between the mucosal barrier and the immune system is crucial for comprehending the mechanisms underlying mucosal immunity [9]. B cells, along with their immunoglobulin A (IgA)+ plasma cell counterparts, are instrumental in preserving mucosal homeostasis and providing a robust defense against pathogenic incursions [10]. Secretory IgA (sIgA), which is uniquely produced by plasma cells (PCs) within the lamina propria, plays a crucial role in mucosal immunity [11]. It enhances barrier protection against enteric pathogens by binding to surface molecules on these pathogens and neutralizing their toxins [12].

In this study, utilizing Fgl2 knockout (KO) mice and bioinformatics approaches, we explored the role of Fgl2 in mucosal immunity and sought to identify the molecular mechanisms by which Fgl2 deficiency regulates B cell activation and fate.

## 2 Result

### 2.1 Fgl2 Deficiency Enhances Intestinal Mucosal Immunity Against Infection

Initial immunofluorescence staining surprisingly showed that Fgl2 expression was decreased while IgA expression was increased in the spleens of *Trichinella spiralis*‐infected mice, relative to uninfected controls (Figure S1a). Furthermore, ELISA analysis of plasma from infected and uninfected mice corroborated these findings, revealing lower levels of Fgl2 and higher levels of IgA in the infected group (Figure S1b,c). Collectively, these results suggest that Fgl2 and IgA play important roles in the host response against intestinal *T. spiralis* infection.

**FIGURE 1:**
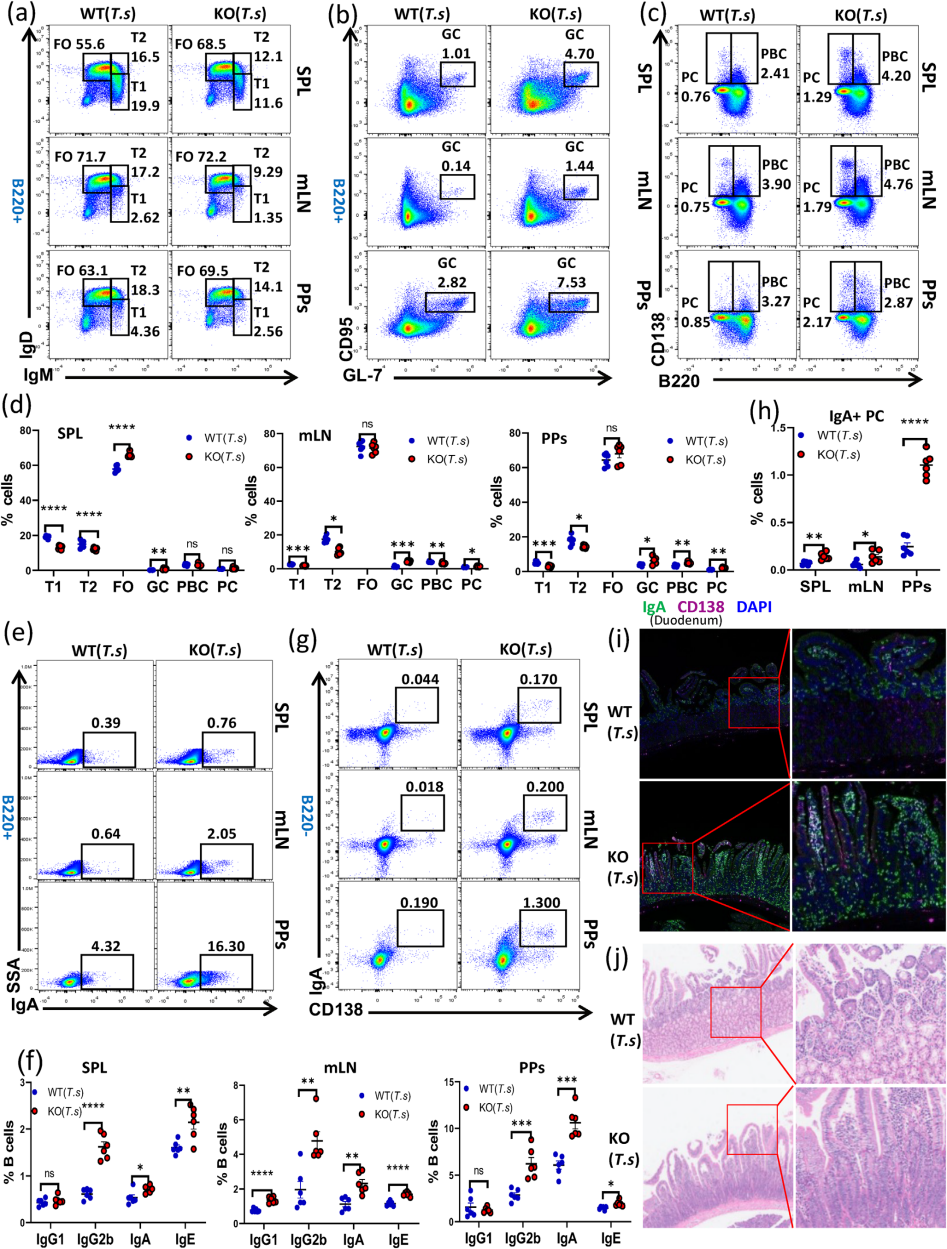
Fgl2 deficiency enhances intestinal mucosal immunity against infection. Intestinal trichinella infection stage: Larvae were harvested from the muscles of mice infected with Trichinella for more than 45 days and were administered via gavage to WT (*T. s*) and KO (*T. s*) mice at a density of 200–400 larvae per 10–200 µL volume. After 5–7 days of infection, mice were euthanized, and spleen, mLN, PPs, and duodenum were collected. (a–h) Flow cytometric analysis of B cell subsets including follicular (FO), transitional 1 (T1), transitional 2 (T2), germinal center (GC) B cells, plasmablasts (PBC), plasma cells (PC), IgA^+^ B cells, and IgA^+^ plasma cells—in the spleen, mesenteric lymph nodes (mLN), and Peyer's patches (PPs) from *Trichinella spiralis*‐infected WT and KO mice. Representative dot plots and the mean percentages of each subset in the spleen are shown (*n* = 5 per group). (i) Immunofluorescence analysis of IgA and CD138 deposition in duodenum tissue sections (green staining for IgA, purple staining for CD138). Shown are representative duodenum and lung sections using a ×60 objective. Scale bar, 10 µm. (j) Hematoxylin and eosin (HE) staining of duodenum sections from WT and KO infected mice. Take the duodenum of the mice that have been infected for 7 days. Shown are representative duodenum using a ×60 objective. Scale bar, 25 µm. Error bars represent the mean (±SD). *p < 0.05, **p < 0.01, ****p < 0.0001, ns: no significant difference.

Flow cytometry was then used to assess the differentiation of B cells in the spleen, mesenteric lymph nodes (mLN), and Peyer's patches (PPs) of WT and KO mice postinfection. Results revealed that the frequency of T1 and T2 B cells in the SPL, mLN, and PPs was reduced in KO mice, while the frequency of FO B cells was significantly increased in the SPL (Figure 1a,d). Additionally, the frequencies of GC B cells and PCs were markedly elevated in the SPL, mLN, and PPs, and the population of plasmablast B cells (PBCs) was also increased in mLN and PPs (Figure 1b–d). We also observed a substantial increase in follicular helper T cells (Figure S1d,e) and IL‐10+ regulatory B cells (Figure S1f,g) within the SPL, mLN, and PPs of *T. spiralis*‐infected KO mice, which are integral to B cell differentiation regulation. Furthermore, examination of the expression of IgG1, IgG2b, IgA, and IgE in the SPL, mLN, and PPs revealed that in KO mice, IgG1 was significantly increased only in the mLN, while IgG2b, IgA, and IgE were significantly increased across the SPL, mLN, and PPs (Figure 1e,f; Figure S2s). These findings suggest that Fgl2 deficiency not only promotes IgA class switching to combat mucosal infection but also induces IgE class switching, potentially participating in allergic reactions caused by *T. spiralis*.

A significant increase in IgA+ PCs was observed in the SPL, mLN, and PPs of *T. spiralis*‐infected KO mice (Figure 1g,h). Immunofluorescence also revealed increased expression of IgA+ PCs and enhanced production of sIgA in the mucosal region of the duodenum in KO mice (Figure 1i). Based on the regulatory role of Fgl2 in mucosal immunity, we noted that KO mice did not exhibit mucosal edema and damage or the shallowing of Lieberkühn's crypts typically seen after *T. spiralis* infection (Figure 1j), and the worm burden in the duodenum was significantly reduced (Figure S1h).

Additionally, we investigated the role of Fgl2 in regulating B cell homeostasis during the muscle stage within five weeks post *T. spiralis* infection. Results revealed that splenic GC B cells in KO mice had collapsed (Figure S2b,f), with no significant difference in IgG1, IgG3, and IgE class switching, but a significant reduction in IgG2b and IgA class switching (Figure S2e,i). The number of PCs and IgA+ PCs in KO mice continued to increase (Figure S2c,d,g,h). Additionally, when comparing the number of larvae in the muscles of the mice, a reduced number of larvae was observed in KO mice compared with the WT group (Figure S2j).

In conclusion, these results indicate that Fgl2 deficiency enhances mucosal immunity against *T. spiralis* infection. Although Fgl2 deficiency aids in the formation of GC B cells and the regulation of immunoglobulin isotype switching during the intestinal phase of *T. spiralis* infection, this effect is not sustained into the muscle phase, leading to the collapse of GC B cells and a significant reduction in Ig isotype switching.

### 2.2 Fgl2 Deficiency Leads to Abnormal Splenic Immune Profiling and B Cell Immunoglobulin Heavy Constant Alpha 1 Clonal Heterogeneity

**FIGURE 2:**
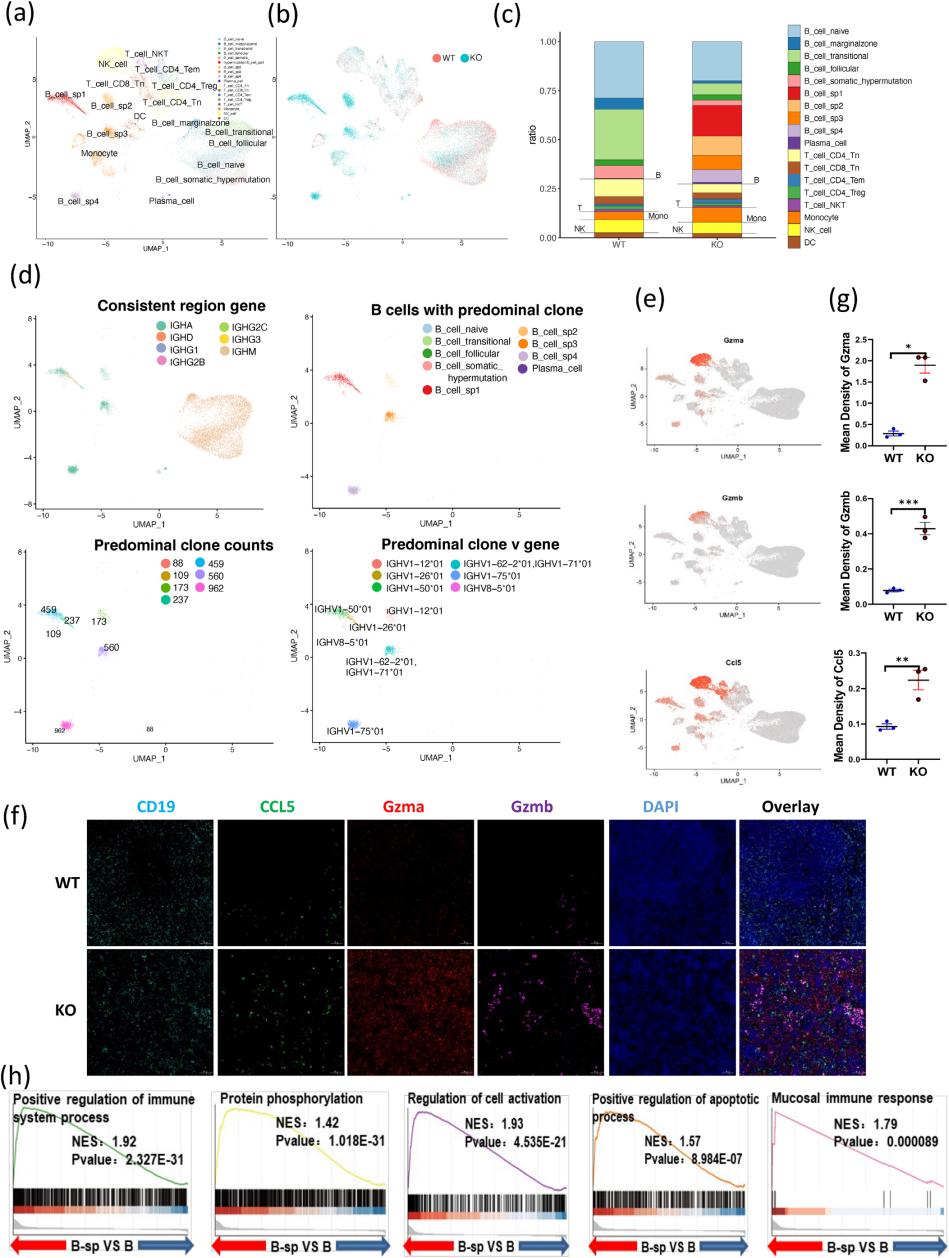
Fgl2 deficiency leads to abnormal splenic immune profiling and B cell immunoglobulin heavy constant alpha 1 (IGHA) clonal heterogeneity. (a) Uniform manifold approximation and projection (UMAP) plots of Single‐cell sequencing of WT and KO splenic B cell subsets. Each dot represents a single cell. (b) UMAP plot highlighting the cellular composition, showing an expansion of specific subsets in KO mice (blue), primarily B cell_sp1, B cell_sp2, B cell_sp3, B cell_sp4, and plasma cells, compared with WT mice. (c) Bar plot showing differences in cell subsets between KO and WT mice. (d) UMAP displaying B cell_sp1, B cell_sp2, B cell_sp3, and B cell_sp4 as B cells with genes in the cell‐constant region, the v gene of the predominant clones, the genes in a predominant B cell clone, and the counts of the predominant B cell clone subsets (scBCR‐seq data). (e) UMAP feature plots showing the expression of selected marker genes (*Gzma*, *Gzmb*, *Ccl5*) across cell subsets, with expression enriched in the B cell_sp1‐4 clusters. (f) Immunofluorescence analysis was conducted on the deposition of Gzma, Gzmb, and Ccl5 in the spleen tissues of WT and KO mice. Shown are representative spleen sections using a ×60 objective. Scale bar, 10 µm. (g) Using Image‐Pro Plus 6.0 (Media Cybernetics, Inc., Rockville, MD, USA), each photo was analyzed to obtain the cumulative optical density value (IOD) of each photo as well as the pixel area of the tissue (AREA). The average optical density value (mean density) was also calculated (WT *n* = 3, KO *n* = 3). (h) Gene set enrichment analysis (GSEA) analysis of B cell_sp1, B cell_sp2, B cell_sp3, and B cell_sp4 subsets, compared with WT B cells.

To investigate how Fgl2 regulates B cells, we conducted single‐cell RNA sequencing (scRNA‐seq) analysis on splenocyte suspensions from 8‐week‐old WT and KO mice to study the direct impact of physiological Fgl2 on B cells. The transcriptional profiling identified 18 cell types, including 9 B cell subsets, 5 T cell subsets, plasma cells, monocytes, natural killer (NK) cells, and Dendritic cells (DC). Compared with the WT group, the KO group showed reduced proportions of naïve B cells, transitional B cells, marginal zone B cells, follicular B cells, CD4‐T naïve, CD8‐T naïve, CD4‐T regulatory, NKT, NK, and DC subsets, while PCs, CD4‐Tem, and monocyte subsets were increased. Notably, we identified four novel B cell subsets in the KO group that could not be classified into classical B cell subsets, and thus named as B cell_sp1, B cell_sp2, B cell_sp3, and B cell_sp4 (Figure 2a–c; Figure S3a). These results indicate that the absence of Fgl2 significantly affects immune cells profiling, particularly B cells.

Subsequently, we performed pairwise analysis between scRNA‐seq and single‐cell B cell receptor sequencing (scBCR‐seq). In immunological and BCR analysis, a “dominant clone” refers to a single clone or genetic variant that accounts for more than 1% of the entire BCR repertoire. The B cell_sp1 subset exhibited the dominant clones immunoglobulin heavy constant alpha 1 (IGHA), IGHM, and IGHD in the constant region, while the other three distinct subsets and PCs only had the dominant clone IGHA in the constant region. This suggests that IGHA clones are the most abundant and dominant in KO B cells. Furthermore, we detailed the number of different BCR heavy chain genes and related VH gene segments in the KO‐specific B cell subsets (Figure 2d). The findings indicated that Fgl2 deficiency leads to B‐cell abnormalities and IgA clonal heterogeneity. Concurrently, we found that B cells exhibiting IGHA clonal heterogeneity highly expressed the genes *Ccl5*, *Gzma*, and *Gzmb* (Figure 2e). To further validate this, multiplex immunofluorescence staining confirmed the presence of B cells with high expression of CCL5, GZMA, and GZMB in the spleens of KO mice (Figure 2f,g). Recent studies have revealed that granzymes GZMA and GZMB are not exclusively secreted by CTL/NK cells; certain B cell subsets can also express them, playing roles in B cell development, activation, antibody regulation, and immunopathology [13]. Ccl5, meanwhile, has been demonstrated to mediate the temporal recruitment and activation of leukocytes, enhance adaptive mucosal, humoral, and cellular immunity, thereby mitigating chlamydial infection [14].

Additionally, we found that KO‐specific B cell subsets highly expressed genes, such as *Ccl4*, *Cxcl2*, *Cxcl10*, *Slpi, S100a8, S100a9, Fcer1g*, and *IL‐1β* (Figure S3b). Moreover, Gene set enrichment analysis (GSEA) of KO ‐specific B cells and normal B cells revealed enrichment in pathways, such as positive regulation of immune system process, regulation of cell activation, protein phosphorylation, mucosal immune response, and positive regulation of apoptotic process (Figure 2h), indicating that the activation of KO B cells is involved in humoral and mucosal immune responses. Moreover, these specific B cells also highly express genes, such as *PDCD1, HAVCR2, CTLA4*, and *ICOS* (Figure S3c), suggesting that these B cells are more prone to exhaustion and may not survive long‐term. In conclusion, the absence of Fgl2 results in abnormalities in the splenic immune profile, with a significant emergence of IGHA clonally heterogeneous B cells. B cells highly expressing the effector molecules CCL5, GZMA, and GZMB were also identified in KO mice, which play an important role during infection. This finding underscores the role of Fgl2 in the modulation of mucosal immune responses.

### 2.3 Fgl2 Deficiency Regulates B Cell Differentiation and Adhesion

**FIGURE 3:**
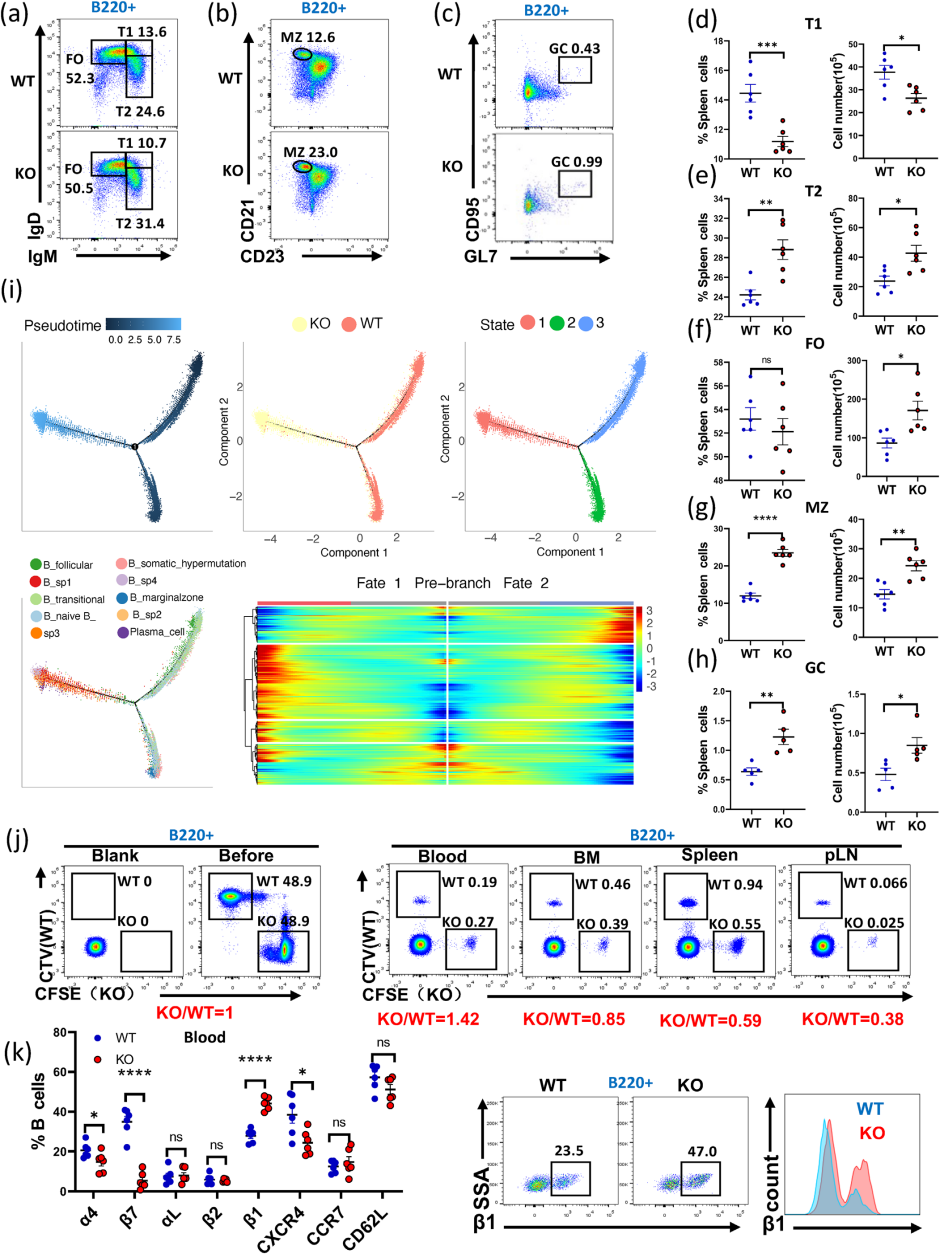
Fgl2 deficiency regulates B cell differentiation and adhesion. (a–h) Flow cytometric analysis of splenic FO, T1, T2, and GC B cell subsets in uninfected (steady‐state), 8‐week‐old WT and Fgl2 KO mice (*n* = 5 per group). Representative plots, along with summary graphs of percentages and absolute numbers, are shown. (i) Pseudotime trajectory analysis revealed 3 branches of splenic B cells, and most B cells in the KO group located on a unique branch, separated from the WT group, at a unique state, and with larger pseudotime values than the WT group. Plasma cells and all specific B cells cluster (B_sp1‐4) were located almost exclusively on the KO unique branch, and the less mature B cells, such as follicular B cells, marginal zone B cells, naïve B cells, transitional B cells, and B cells with somatic hypermutation, were located on the WT branches. The heatmap of the DEGs between KO branch and WT branches showed the difference in gene expression patterns. (j) CTV‐ and CFSE‐labeled WT and KO splenic B cells were mixed in a 1:1 ratio and injected into WT recipient mice. After 3 h, blood, bone marrow (BM), spleen, and peripheral lymph nodes (pLN) cells of recipient mice were stained with anti‐B220‐APC, and the proportions of WT and KO cells were compared. (k) Statistical analysis of adhesion molecules and integrin expression on blood B cells from WT and KO mice, along with representative flow cytometry plots and histograms for β1 integrin (*n* = 5). Data are shown as mean ± SD. Each symbol represents a single mouse. *p < 0.05, **p < 0.01, ns: not significant.

Fgl2 expression in various B‐cell subsets of the spleen from WT mice was analyzed using scRNA‐seq, revealing the following pattern: plasma cells (PC) > marginal zone (MZ) > transitional (T1, T2) ≈ native > follicular (FO) B cells (Figure S3d). Through the examination of spleen B cell subsets by flow cytometry (Figure 3a–c), KO mice had higher proportions and cell numbers of T2, MZ, and GC B cells compared with WT mice (Figure 3e,g,h), whereas the proportion and number of T1 B cells were lower in *Fgl2*‐KO mice (Figure 3d). Although the proportion of FO B cells did not differ between the two groups, the absolute number of FO B cells was higher in KO mice (Figure 3f).

To better understand the effects of Fgl2 on B‐cell differentiation while excluding potential interference from other immune cell types, particularly T cells, a bone marrow chimeric experiment was generated. Irradiated CD45.1 recipient mice were reconstituted with a 1:1 mixture of bone marrow cells from CD45.2 WT or CD45.2 *Fgl2*‐KO mice and CD45.1 WT mice (Figure S4a). In the CD45.2 population, we observed an increase in T2, MZ, and GC B cells and a decrease in T1 B cells, confirming that the B cell differentiation defects in KO mice were intrinsic to Fgl2 deficiency (Figure S4b–h).

Pseudotime trajectory analysis of splenic B cells from KO and WT mice revealed distinct differentiation patterns. Specifically, KO B cells exhibit a more mature stage of differentiation, while WT B cells are more likely in a naïve state (Figure 3i). Additionally, B cell homing assays demonstrated that KO B cells were retained more in peripheral blood, with fewer B cells returning to the bone marrow (BM), spleen (SPL), and peripheral lymph nodes (pLN) (Figure 3j). Flow cytometric analysis of adhesion molecules and integrins on peripheral blood B cells showed reduced expression of α4, β7, and CXCR4 on KO B cells, whereas β1 integrin expression was significantly increased, which is likely the primary factor responsible for B cell retention in peripheral blood (Figure 3k; Figure S5a).

To further investigate the impact of Fgl2 on B cell development and differentiation, we performed flow cytometric analysis of bone marrow B cells. No significant differences were observed between bone marrow cell subsets of KO and WT mice (Figure S5b–d). We also examined the IL‐7 receptor (CD127), which is essential for B cell development, and found no differences in expression levels between WT and KO mice (Figure S5e). Additionally, we observed a reduction in the number of peritoneal B1a cells and an increase in B1b cells in KO mice (Figure S5f–h). In summary, Fgl2 deficiency regulates B cell differentiation and adhesion, thereby modulating B cell function.

### 2.4 Fgl2 Negatively Regulates BCR Signal Transduction and Actin Polymerization

**FIGURE 4:**
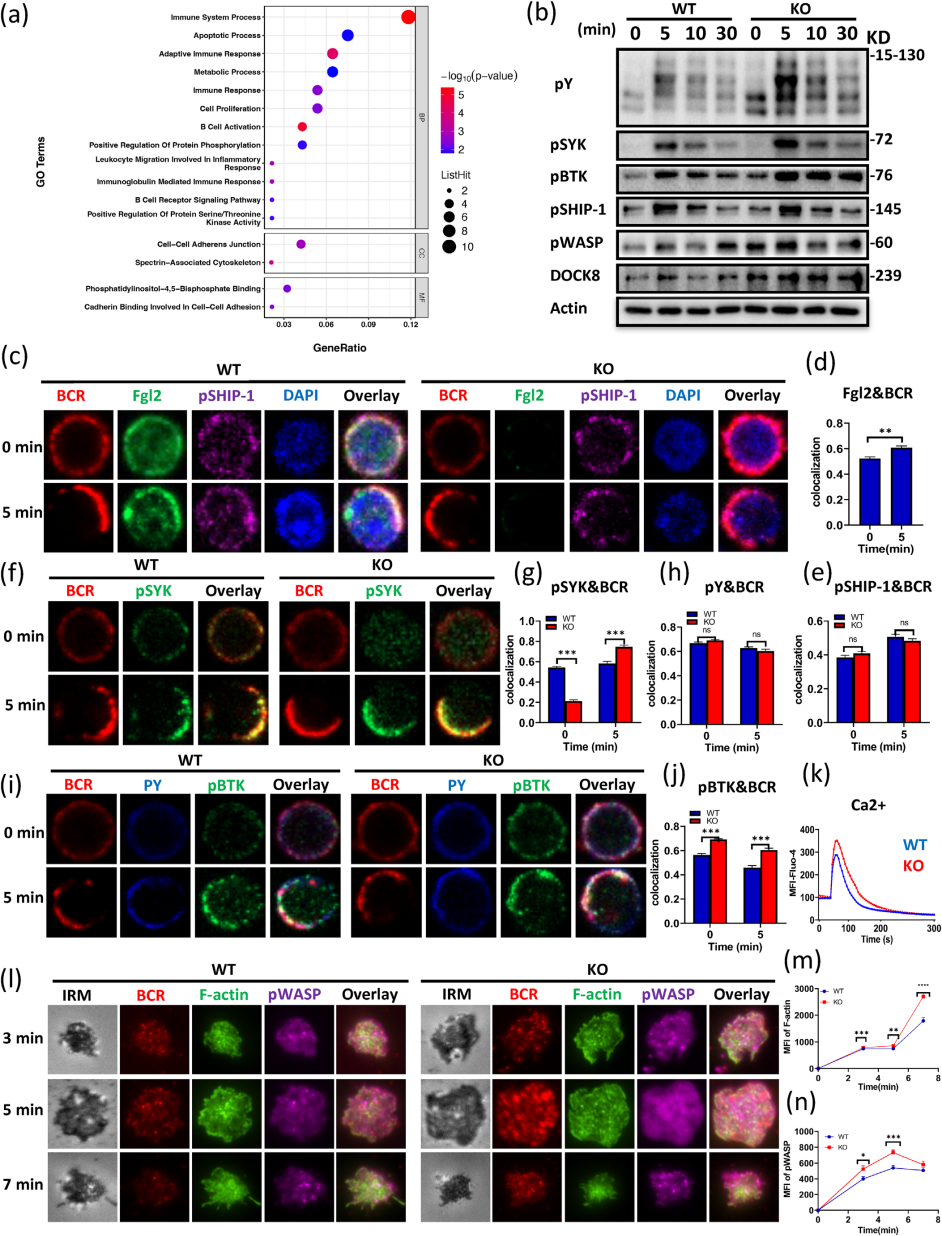
Fgl2 negatively regulates BCR signal transduction and actin polymerization. (a) WT and KO splenic B cells were isolated and cultured for 24 h with F(ab')_2_ anti‐mouse Ig (M + G) and CD40, followed by proteomic and Gene Ontology (GO) analysis. (b) The cells were incubated with biotin‐conjugated F(ab')_2_ anti‐mouse Ig (M + G) and streptavidin, followed by activation at 37°C for 5, 10, and 30 min. Western blot analysis was performed to detect the expression levels of pY, pSYK, pBTK, pSHIP‐1, pWASP, and DOCK8. (c–j) Splenic B cells from WT and KO mice were stimulated with AF594 F(ab')_2_ goat anti‐mouse IgG + IgM (10 µg/mL), then fixed, permeabilized, and stained for Fgl2, pSHIP‐1, pSYK, pY, or pBTK. The colocalization of Fgl2, pSHIP‐1, pSYK, pY, or pBTK with the BCR was analyzed using Pearson's correlation coefficient. Images were captured using a Zeiss confocal fluorescence microscope and analyzed using the NIS‐elements AR 5.01. Scale bars, 2.5 µm. (k) Ca^2+^ flux in splenic B cells from WT and KO mice stimulated with 10 µg/mL biotin‐conjugated F(ab')_2_ anti‐mouse Ig (M + G). Data are representative of three independent experiments. (l) Splenic B cells from WT and KO mice were incubated with AF546‐mB‐Fab‐anti‐Ig tethered to lipid bilayers for 3, 5, and 7 min, then fixed, permeabilized, and stained for pWASP/F‐actin. Shown are representative images captured using Nikon TIRFm from three independent experiments. Scale bars, 2.5 µm. (m, n) The MFI of pWASP, F‐actin in the contact zone were quantified using NIS‐Elements AR 5.0.1 software. Data are shown as mean ± SD. *p < 0.05; **p < 0.01; ***p < 0.001; ****p < 0.0001.

After stimulation with the soluble antigen F(ab')_2_‐anti‐mouse Ig(M+G), BCR induces downstream immune signaling in B cells. WT and KO mouse B cells were isolated and cultured in F(ab')_2_+ anti‐CD40 incubator for 24 h. We compared the proteomic profiles of WT and KO B cells after stimulation and found that Fgl2‐deficient B cells were enriched in immune regulatory pathways (Figure 4a).

Subsequently, the purified splenic WT and KO B cells were stimulated with soluble antigen F(ab')_2_ for 5, 10, and 30 min, and B‐cell signaling was analyzed via western blotting. Results revealed that KO B cells exhibited increased total BCR signaling levels, including pY, pSyk, pBtk, pSHIP‐1, and actin‐related molecules (pWASP, DOCK8), which was consistent with the proteomic pathway analysis (Figure 4b). Furthermore, confocal microscopy (CFm) revealed significant changes in the colocalization of Fgl2 and BCR on B cells after F(ab')_2_ stimulation for 5, 10, and 30 min (Figure 4c,d). Compared with WT B cells, no significant difference in colocalization of pSHIP‐1 and BCR was observed in KO B cells (Figure 4c,e). The colocalization of pSYK and BCR decreased at 0 min but increased at 5 min (Figure 4f,g), and the colocalization of pY and BCR showed no significant difference (Figure 4i,h). The colocalization of pBTK and BCR significantly increased at both 0 and 5 min (Figure 4i,j). Moreover, PLCγ2‐induced calcium (Ca^2^^+^) mobilization, which mediates downstream signaling, was found to be enhanced in KO B cells upon F(ab')_2_ stimulation (Figure 4k).

Next, to investigate the relationship between Fgl2 and actin dynamics in B cells, the activation and localization of WASP, and F‐actin reorganization in WT and KO B cells were examined. Results found that the colocalization of pWASP and BCR, as well as F‐actin and BCR, decreased at both 0 and 5 min (Figure S6a–c). Scanning electron microscopy (SEM) revealed more detailed morphological changes in B cells (Figure S6d), and after F(ab')_2_ stimulation, the number and length of filopodia in KO B cells were significantly increased (Figure S6e). Additionally, B‐cell spreading assays showed that KO B cells exhibited greater spreading ability (Figure S6f,g; the detailed experimental procedures were conducted following established protocols [15]).

Using total internal reflection fluorescence microscopy (TIRFm), which has higher resolution than CFm, we captured spatial images of the moment when B cells contacted membrane antigen F(ab')_2_ (mAg). After mAg stimulation for 3, 5, and 7 min, the F‐actin levels at the contact site of KO B cells were increased compared with WT B cells (Figure 4l,m). Consistent with the increased F‐actin accumulation, pWASP levels in KO B cells also increased at 3 and 5 min after stimulation (Figure 4n). Furthermore, these changes were accompanied by enhanced recruitment of pSyk and pSHIP‐1 to the BCR in KO B cells (Figure S6h–j).

Together, these findings indicate that Fgl2 deficiency enhances the recruitment of WASP‐mediated F‐actin on the plasma membrane, associated with increased BCR aggregation and enhanced BCR signal transduction.

### 2.5 Fgl2‐Rack1 Interaction Regulates B Cell Signaling

**FIGURE 5:**
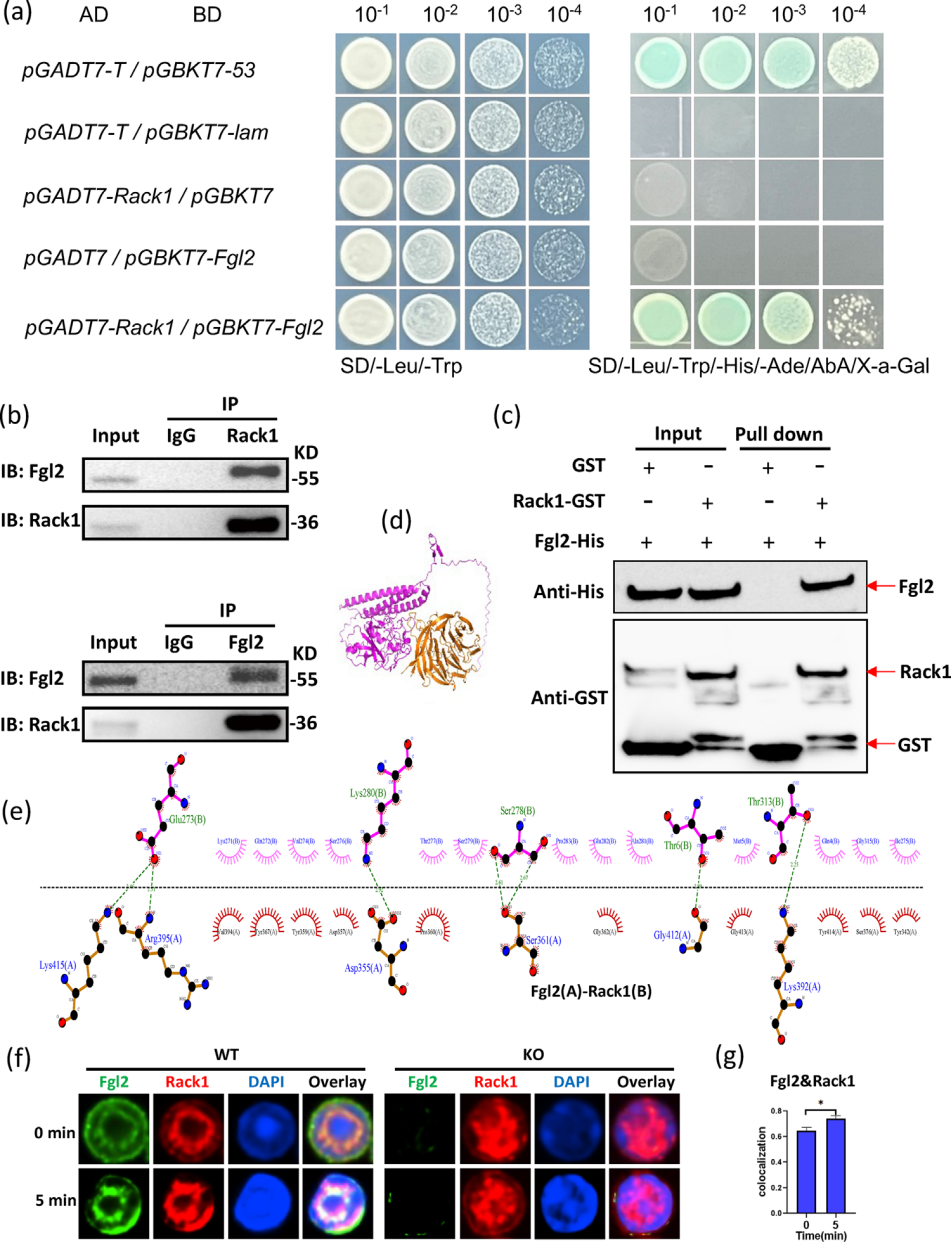
Fgl2‐Rack1 interaction regulates B cell signaling. (a) The *Rack1* gene was constructed into the pGADT7 vector. Using the Y2H system (Takara, Kyoto, Japan), plasmid pGADT7‐Rack1 and pGBKT7 were transferred into yeast strain Y2H Gold and coated on DDO, TDO, and QDO plates for 3–5 days at 30°C. The vector of pGADT7‐Rack1 without self‐activation and toxicity and pGBKT7‐Fgl2‐JD were transferred into the Y2H Gold strain and coated in DDO and QDO plates for 3–5 days at 30°C. The growth of monoclonal colonies on the selection plates confirmed a direct interaction between the two proteins. (b) Co‐immunoprecipitation (CO‐IP) assay was performed using lysates from WT mouse splenic B cells. Anti‐Rack1 antibody (control: rabbit IgG) or anti‐Fgl2 antibody was used for immunoprecipitation. The interaction between endogenous Rack1 and endogenous Fgl2 was detected by western blot. (c) GST pull‐down assay showing the direct interaction between Rack1 and Fgl2. GST alone or GST‐Rack1 fusion protein was incubated with His‐Fgl2 protein, and the interaction was detected by western blot. (d, e) Computational modeling of the Fgl2 and Rack1 interaction. Three‐dimensional structural models of the Fgl2 and Rack1 proteins were generated using AlphaFold3. To investigate the potential interaction between Fgl2 and Rack1, we performed molecular docking simulations, also using AlphaFold3. The optimal predicted structures for both proteins were used as input. The simulation calculated potential binding interfaces by evaluating factors such as surface electrostatic potential, hydrogen bonding possibilities, and hydrophobic interactions to predict the most likely binding mode between Fgl2 and Rack1. (f, g) Splenic B cells from WT and KO mice were stimulated with AF594‐conjugated F(ab′)_2_ goat anti‐mouse IgG + IgM (10 µg/mL) for 0 and 5 min, then fixed, permeabilized, and stained for Fgl2 and Rack1. Co‐localization between Fgl2 and Rack1 was quantified using the Pearson correlation coefficient. Shown are representative blots from three independent experiments. *p < 0.05; **p < 0.01; ***p < 0.001; ****p < 0.0001.

To identify protein interactors of Fgl2 in B cells, we performed a yeast two‐hybrid (Y2H) screen. A truncated Fgl2 construct, designated Fgl2‐JD (lacking the N‐terminal signal peptide to ensure nuclear localization), was used as bait to screen a B‐cell cDNA library. This screen identified 58 candidate interacting proteins. Based on Gene Ontology annotation and pathway analysis of these candidates, we prioritized Rack1 for further investigation due to its established role in immune cell signaling. The direct interaction between Fgl2 and Rack1 was subsequently validated (Figure 5a). Furthermore, the interaction was validated in WT mouse‐purified B cells using co‐immunoprecipitation (Co‐IP) experiments (Figure 5b). To further probe the direct nature of the Fgl2 and Rack1 interaction, GST pull‐down assays were performed. We utilized bacterially expressed GST‐Rack1 and His‐Fgl2. The results demonstrated that GST‐Rack1, but not GST alone (as a negative control), bound to His‐Fgl2, confirming a direct interaction between Fgl2 and Rack1 (Figure 5c). To gain deeper insights into the interaction sites between Fgl2 and Rack1, three‐dimensional (3D) structural modeling of Fgl2 and Rack1 was performed using AlphaFold3. Molecular docking simulations were conducted as well, and the results revealed that important hydrogen bonds were formed between Fgl2 Lys415 and Arg395 and Rack1 Glu273. Additional hydrogen bonds were also detected between Fgl2 Asp355 and Lys280, Ser361 and Ser278, Gly412 and Thr6, as well as Lys392 and Thr313. The lengths of these hydrogen bonds ranged from 2.46 to 2.89 Å, indicating their crucial role in the formation and stability of the protein complex. The presence of these hydrogen bonds, particularly involving amino acid residues with polar side chains such as lysine (Lys), arginine (Arg), aspartic acid (Asp), and serine (Ser), underscores their importance in mediating protein‐protein interactions (Figure 5d,e).

To further investigate the interaction between Fgl2 and Rack1, we examined their subcellular localization in splenic B cells using confocal microscopy. In WT B cells, we observed significant colocalization between Fgl2 and Rack1, which was further enhanced upon BCR stimulation with anti‐IgG/IgM for 5 min (Figure 5f). This finding, combined with our previous observation of Fgl2's recruitment to the BCR, suggests that the Fgl2–Rack1 interaction plays a role in BCR signal transduction. Moreover, we made a key discovery regarding how Fgl2 intrinsically regulates Rack1's spatial organization. In both resting and activated Fgl2‐sufficient B cells, Rack1 displayed a distinct ring‐like distribution surrounding the nucleolus. In contrast, this organized ring structure was lost in Fgl2‐deficient B cells; instead, Rack1 exhibited a diffuse, punctate distribution within the nucleus (Figure 5g). In conclusion, the interaction between Fgl2 and Rack1 is involved in BCR signaling, and the detailed mechanisms of these interactions not only reveal how Fgl2 and Rack1 form stable complexes through hydrogen bonding but also provide important structural insights into their roles in cellular signal transduction and biological functions.

### 2.6 Fgl2 Regulates B Cell Metabolism and Differentiation by Inhibiting Rack1‐Mediated AKT Activation

**FIGURE 6:**
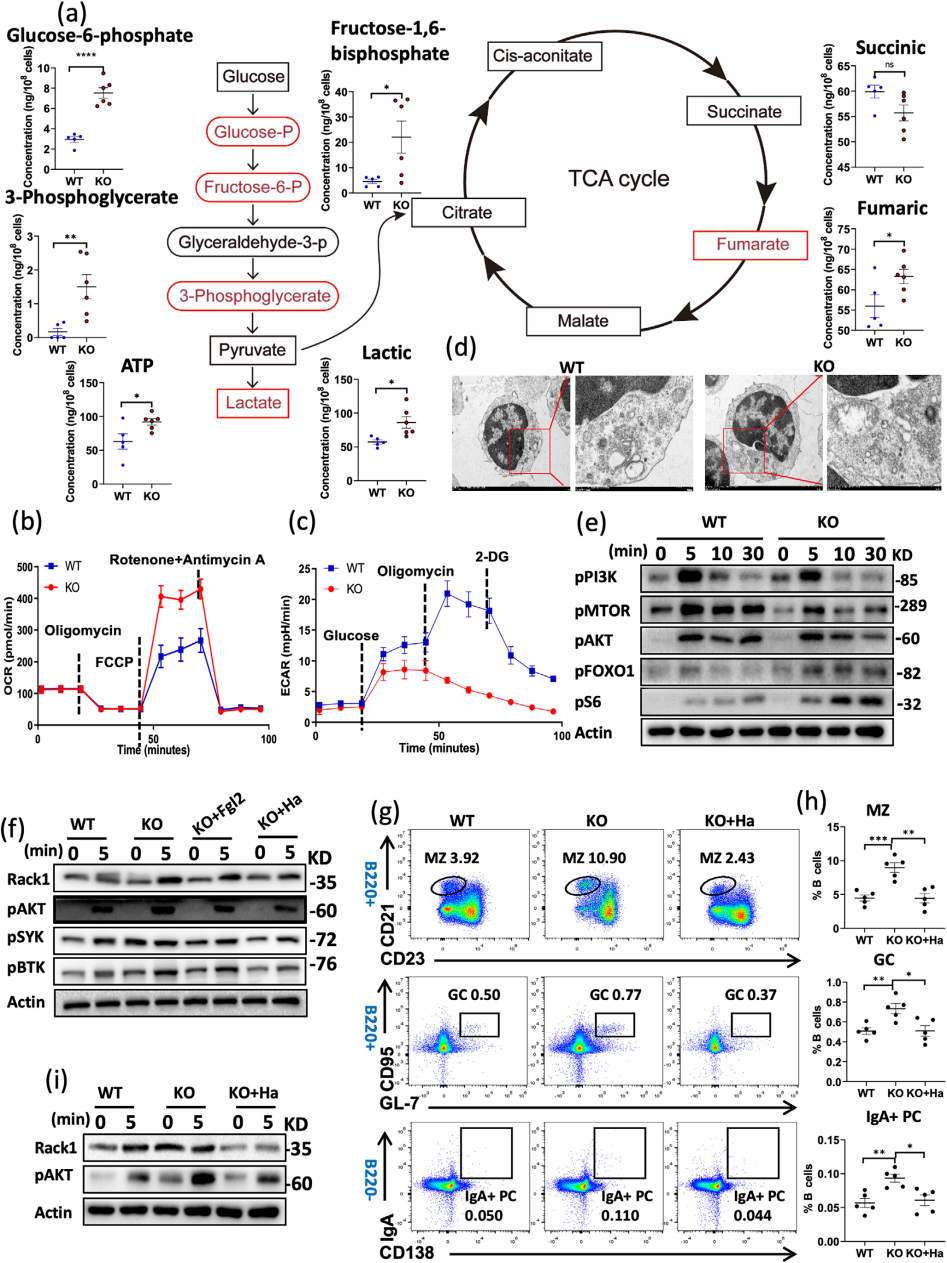
Fgl2 regulates B cell metabolism and differentiation by inhibiting Rack1‐mediated AKT activation. (a) WT and KO splenic B cells were isolated and cultured for 24 h in the presence of F(ab')_2_ anti‐mouse Ig (M + G) and CD40, followed by comparison of their metabolic products and metabolomic analysis (WT *n* = 5, KO *n* = 6). (b) Oxygen consumption rate (OCR) measurement in WT and KO B cells to assess mitochondrial respiration. (c) Extracellular acidification rate (ECAR) measurement in WT and KO B cells to evaluate glycolytic activity. (d) Transmission electron microscopy (TEM) analysis of subcellular structures, including mitochondria, in WT and KO B cells after 24 h of culture in the presence of F(ab')_2_ anti‐mouse Ig (M + G) and CD40. (e) Western blot analysis of phosphorylated proteins (pPI3K, pMTOR, pAKT, pFOXO1, pS6) in splenic B cells activated with biotin‐conjugated F(ab')_2_ anti‐mouse Ig(M+G) and streptavidin. (f) The spleen B cells of KO mice were pretreated with 20 µM Rack1 inhibitor (Harringtonolide) and 10 ng Fgl2 protein for 2 h, and then stimulated with sAg(F(ab')_2_ anti‐mouse Ig(M+G)) for 5 min together with the B cells of WT mice. Subsequently, the levels of Rack1, pAKT, pSYK, and pBTK were detected by western blotting. (g, h) At 8 weeks of age, WT and KO mice were intraperitoneally injected with 0.5 µg/g of physiological saline or Rack1 inhibitor twice a week for 28 days, and then MZ, GC B cells, and IgA+ plasma cells were analyzed by flow cytometry (WT *n* = 5, KO *n* = 5). (i) At 8 weeks of age, WT and KO mice were intraperitoneally injected with 0.5 µg/g of physiological saline or Rack1 inhibitor twice a week for 28 days. Splenic B cells were collected and stimulated with sAg (F(ab')_2_ anti‐mouse Ig(M+G)). Western blotting was used to detect the levels of Rack1 and pAKT. Data are presented as mean ± SD. *p < 0.05, **p < 0.01, ****p < 0.0001, ns: not significant.

The main subsequent effect of BCR activation is metabolic activity. After the same stimulation for proteomic analysis (F(ab')_2_ + anti‐CD40 for 24 h), the metabolomic profiles of splenic B cells in WT and KO mice were compared with assess the impact of Fgl2 on B cell metabolic adaptability. Notably, the levels of glycolytic products, such as glucose‐6‐phosphate, fructose‐6‐phosphate, 3‐phosphoglycerate, and lactic acid, were elevated in KO B cells. In the tricarboxylic acid (TCA) cycle, there were no differences in succinate levels, while fumarate levels were increased (Figure 6a). In addition, following stimulation with F(ab')2‐Ig (M + G), seahorse assay showed that KO B cells had higher ATP production levels and maximal respiratory capacity than WT B cells; however, the glycolysis ability of KO B cells was decreased (Figure 6b,c). Furthermore, transmission electron microscopy (TEM) revealed obvious mitochondrial swelling and an increase in ribosomes in both WT and KO activated mouse B cells (Figure 6d), indicating enhanced cellular activity, which is consistent with the increase in ATP production and metabolic activity.

Previous studies have indicated that the elevation of Rack1 promotes the phosphorylation of AKT, thereby influencing metabolism [16]. Consequently, we examined the AKT‐mTOR metabolic signaling in B cells. We observed a reduction in the proximal phosphorylation of PI3K (p‐PI3K) and mTOR (p‐mTOR) in KO B cells. However, the levels of phosphorylated AKT (p‐AKT), phosphorylated S6 (p‐S6), and phosphorylated FOXO1 (p‐FOXO1) were paradoxically increased (Figure 6e). These findings suggest that AKT activation might be a critical nexus in the pathway through which Fgl2 regulates B cell function. To investigate whether Fgl2 dictates B cell fate via Rack1 and subsequent AKT activation, both in vitro and in vivo experiments were conducted. B cells were purified and pretreated with either recombinant Fgl2 protein or the Rack1 inhibitor Harringtonolide (Ha) before antigen stimulation. Further Western blot analysis revealed that, compared with untreated KO B cells, the addition of recombinant Fgl2 exerted influence on the activation of Rack1, p‐AKT, and p‐BTK. However, this inhibitory effect was considerably less pronounced than that observed with the Rack1 inhibitor (Ha) (Figure 6f). Concurrently, in vivo experiments were conducted where KO mice received intraperitoneal injections of the Ha every other day, while WT and KO mice received saline. After 28 days, splenic B cell differentiation was assessed. Compared with saline‐treated KO mice, Ha‐treated KO mice exhibited significantly reduced proportions of MZ B cells, GC B cells, and IgA+PC (Figure 6g,h). Furthermore, Western blot analysis of purified splenic B cells from these mice showed markedly lower levels of Rack1 and p‐AKT in the Ha‐treated group compared with the saline‐treated KO group (Figure 6i). Therefore, these findings suggest that Fgl2 influences B cell metabolism and differentiation by inhibiting Rack1‐mediated AKT activation.

### 2.7 Fgl2 Regulates B Cells in Patients with Mucosal Immune Conditions by Inhibiting Rack1‐Mediated AKT Activation

**FIGURE 7:**
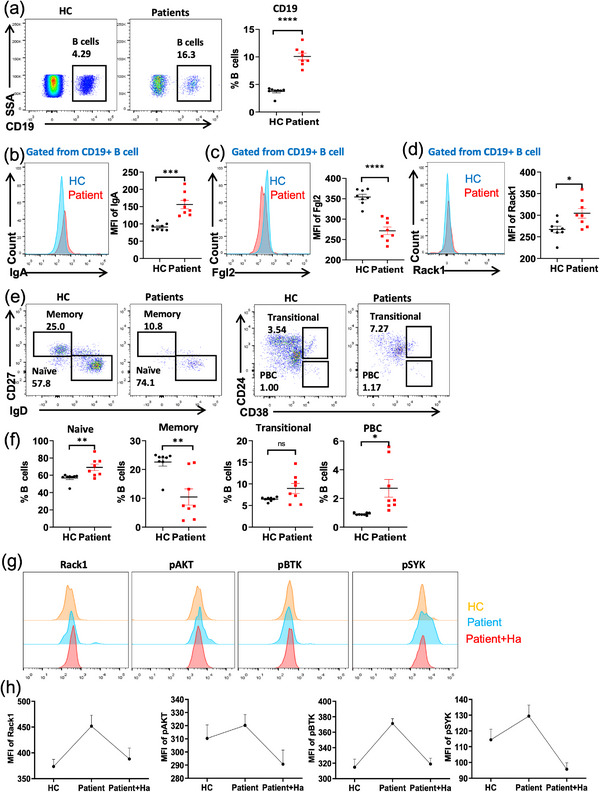
Fgl2 regulates B cells in patients with mucosal immune conditions by inhibiting Rack1‐mediated AKT activation. (a) Flow cytometric analysis of the proportion of B cells in peripheral blood from healthy controls (HC, *n* = 9) and COVID‐19 patients (patient, *n* = 8). (b–d) Flow cytometric histograms and mean fluorescence intensity (MFI) of IgA, Fgl2, and Rack1 expression on B cells in healthy controls and COVID‐19 patients. (e, f) Flow cytometric analysis of B cell subsets in PBMCs from HCs and patients. Representative flow cytometric plots of memory B cells, Naïve B cells, transitional B cells, and plasma B cells in the peripheral blood of healthy controls and COVID‐19 patients. (g, h) The patient's PBMCs were first pretreated with 20 µM Rack1 inhibitor (Harringtonolide) for 2 h, and then stimulated with human sAg for 5 min. Phosphorylation flow cytometry was used to detect the expression levels of Rack1, pAKT, pBTK, and pSYK in B cells (patient, *n* = 6). Data are presented as mean ± SD. *p < 0.05, **p < 0.01, ****p < 0.0001, ns: not significant.

Subsequently, to validate whether Fgl2 in B cells regulates their differentiation and function via Rack1 and AKT phosphorylation in the context of human mucosal immunity, we selected patients with the early stage of COVID‐19 infection as a clinical cohort (Table S1). The levels of Fgl2 in B cells of COVID‐19 patients were investigated to elucidate its role in the mucosal immune response. Flow cytometry analysis revealed that the percentage of B cells in the peripheral blood of patients with COVID‐19 was significantly higher than in healthy individuals (Figure 7a). Additionally, an upregulation of IgA expression and a downregulation of Fgl2 within B cells from infected patients were observed (Figure 7b,c), corroborating the results from our animal model studies. Furthermore, we observed an upregulation of Rack1 expression in B cells from infected patients (Figure 7d), suggesting that Fgl2 and Rack1 play a role in regulating mucosal immunity in COVID‐19 patients. In addition, we conducted a comprehensive analysis of various B cell subsets, including naïve B cells, memory B cells, transitional B cells, and PBCs. Our results indicated that, compared with healthy individuals, the levels of all B‐cell subsets were elevated in COVID‐19‐infected patients, with the exception of memory B cells, which were significantly reduced (Figure 7e,f).

To validate whether the Fgl2‐Rack1 interaction regulates BCR signaling in B cells from patients with mucosal immune conditions, we stimulated PBMCs from healthy donors, COVID‐19 patients, and COVID‐19 patient PBMCs pretreated with the Rack1 inhibitor (Ha) using F(ab')2 fragments. Results revealed that, compared with B cells from healthy donors, B cells from infected patients exhibited significantly increased levels of Rack1, p‐AKT, p‐BTK, and p‐SYK upon stimulation. Pretreatment of patient PBMCs with Ha resulted in reduced levels of p‐AKT, p‐BTK, and p‐SYK following stimulation when compared with untreated patient PBMCs (Figure 7g,h). These results indicate that, during the mucosal immune response, Fgl2 regulates B cell differentiation and function, at least in part, by inhibiting Rack1‐mediated AKT activation.

## 3 Discussion

The regulatory role of Fgl2 in B cell biology remains incompletely understood. This study advances our knowledge by comprehensively characterizing the phenotypic and functional alterations in B cells following Fgl2 deficiency, an investigation spurred by our initial observation that Fgl2 deficiency promotes the differentiation of murine B cells into IgA+ plasma cells, thereby enhancing resistance to intestinal *Trichinella spiralis* infection. A key novel finding was the identification of distinct B cell subsets in KO mice, predominantly characterized by IGHA clonal dominance and high expression of *Ccl5*, *Gzma*, and *Gzmb*. Furthermore, we established a direct interaction between Fgl2 and Rack1, through which Fgl2 modulates B cell metabolism and differentiation by suppressing AKT phosphorylation. Importantly, this regulatory axis was conserved in humans; in B cells from patients with the early stage of COVID‐19 infection, Fgl2 similarly regulated B‐cell activation and differentiation by inhibiting Rack1‐mediated AKT phosphorylation. Collectively, these results elucidate a critical pathway wherein Fgl2, by inhibiting Rack1‐dependent AKT activation, governs B‐cell participation in mucosal immunity.

The majority of studies conducted on the intestinal mucosa have focused on PPs and mLNs, and the understanding of the intestinal immune response under *T. spiralis* infection remains incomplete. Moreover, research on B lymphocytes in mLN and PPs is rare. Consequently, our investigation into the mucosal immune response regulated by Fgl2 has been enriched by including assessments of the SPL, mLN, and PPs, thereby broadening the scope of research in this field. Our results revealed that Fgl2 deficiency induces a roughly consistent B‐cell homeostasis in the SPL, mLNs, and PPs, with the exception of differences in follicular B cells and PCs. Studies have suggested that B cells play a unique role in mucosal protection, indicating the existence of a functional gut‐SPL axis. Combining our findings from the intestinal lymph nodes and SPL, it is inevitable that Fgl2 regulation of mucosal immunity is closely related to the gut–SPL axis [17]. Additionally, research has indicated that during *Salmonella typhimurium* infection, the recruitment of Sca‐1 monocytes to lymphoid organs disrupts pre‐existing GC responses. GC responses induced by influenza, malaria parasites, or commensal bacteria are exacerbated following *S. Typhimurium* infection [18]. In our study, we observed the collapse of GCs five weeks post *T. spiralis* infection. Beyond the exhaustion and metabolic aspects associated with overactivation of B cells, whether this is a direct effect of B cells or involves other cells and cytokines remains to be further explored.

Our findings revealed a significant increase in MZ B cells in flow cytometry results following the absence of Fgl2, consistent with the notion that Fgl2 deficiency aids in B cell differentiation and activation. However, scRNA‐seq analysis indicated a reduction in the proportion of MZ B cells under Fgl2‐deficient conditions. Concurrently, we identified four novel B cell subpopulations in KO B cells and attempted to trace their origins. Comparing the changes in the overall cellular subpopulations, these new subpopulations are likely derived from the differentiation or alteration of MZ B cells, adapting to the complex immune environment. This discovery provides direction for our subsequent in‐depth research.

Gzmb has traditionally been considered an exclusive effector molecule of NK cells and cytotoxic T lymphocytes (CTLs) [19, 20]. However, recent investigations have revealed that a combination of IL‐21 stimulation and BCR engagement can empower B cells to produce and secrete an active form of Gzmb [21]. These B cells, endowed with cytotoxic potential, are gaining increasing attention for their diverse roles in immunity. A particularly striking finding in our study was the elevated expression of both *Gzmb* and *Gzma* in the clonally heterogeneous IgA+ B cells identified in the spleens of KO mice, notably in the apparent absence of exogenous IL‐21 and strong, overt BCR co‐stimulation typically required for inducing granzyme expression in B cells. This observation implies that the functional repertoire of these Fgl2‐deficiency‐associated IgA+ B cells may be broader and more significant than currently appreciated. Specifically, these GzmA/GzmB‐expressing B cells in KO mice could exert direct cytotoxic functions, potentially playing a crucial and hitherto unrecognized role in host defense against various infections and in antitumor immunity.

In conclusion, Fgl2 regulates the biological activity of B cells, influencing differentiation and metabolism, while also playing a role in the modulation of mucosal immunity. Additionally, Fgl2 interacts with Rack1 and participates in the regulation of BCR signaling. As an important immune regulatory molecule, Fgl2 holds significant potential as a target for drug development within the corresponding signaling pathways, offering promise for the treatment of immune‐related diseases. Furthermore, Fgl2 is also a crucial therapeutic target in the contexts of infectious immunity and mucosal immunity.

## 4 Materials and Methods

### 4.1 Mice

*Fgl2‐*KO mice (C57BL/6J background, male) were generated by Shanghai Model Organisms Center, Inc. (Shanghai, China). C57BL/6J mice (CD45.2 and CD45.1) (male, 8 weeks old, 20–24 g) were purchased from Charles River (Beijing, China). Wild‐type (WT) C57BL/6J mice (male, 8 weeks old, 20–24 g) were purchased from Shulaibao Biotechnology Co., Ltd. (Wuhan, China). All mice were housed in the specific‐pathogen‐free (SPF) experimental animal facility at Tongji Medical College, Huazhong University of Science and Technology (ethics approval reference number: 4900).

### 4.2 Blood Samples From Patients

PBMCs were isolated from peripheral blood samples collected from eight patients exhibiting upper respiratory tract infection symptoms who were confirmed SARS‐CoV‐2 positive during the acute phase of COVID‐19. For comparison, PBMCs were also isolated from peripheral blood collected from eight healthy control individuals. All procedures involving human participants were reviewed and approved by the Medical Ethics Committee of Tongji Hospital, Tongji Medical College, Huazhong University of Science and Technology (ethical approval no. S086). Written informed consent was obtained from all participants before sample collection.

### 4.3 Flow Cytometry

Following incubation with Fc blocker anti‐CD16/CD32 (101319, BioLegend), splenic mononuclear cells (2 × 10^6^), bone marrow cells (2 × 10^6^), or peritoneal cavity cells (1 × 10^6^) were stained with antibodies for 30 min.

For PBMCs from COVID‐19 patients with respiratory symptoms, 2 µg/mL polyclonal F(ab')_2_ rabbit anti‐human IgM (Jackson ImmunoResearch, West Grove, PA, USA) and 0.05 µg/mL recombinant‐human CD40L (Alexis Biochemicals, SanDiego, CA, USA) were incubated with the cells in a 37°C incubator for 24 h. Cells were fixed, permeabilized, and then stained with antibodies. All flow cytometry data were collected using a flow cytometer (BD Biosciences), and data were analyzed using FlowJo software. Antibodies used in this study are listed in Table S2.

### 4.4 Western Blotting

Purified splenic B cells were incubated with biotinylated F(ab')_2_‐anti‐mouse Ig (M + G) (10 µg/mL) on ice for 30 min, followed by streptavidin 5 (20 µg/mL) for an additional 10 min. Subsequently, the cells were activated at 37°C in a water bath for 5, 10, and 30 min and lysed using RIPA buffer containing a protease inhibitor cocktail, NaF, and Na3VO3. Cell lysates were analyzed by SDS‐PAGE and Western blot with antibodies. Immunoreactive bands were captured with a ChemiDocXRS + imaging system. Antibodies used were detailed in Table S2.

### 4.5 TIRFm and Confocal Microscopy

The detailed experimental procedures were conducted following established protocols [22, 23].

### 4.6 Calcium Inflow Confocal Microscopy

Purified 2 × 10^6^ B cells were labeled with Fab‐647 and Fluo‐4AM, added to the chamber precoated with polylysine, and cultured for 30 min in a 37°C water bath. Once the visual field was identified using Digital Image Correlation, 280 µL of preheated calcium‐containing extracellular fluid was added into the chamber for filming. After 30 s of filming, the stimulant F(ab')_2_ (2.5 µg/mL) was added immediately and photographed for 5 min.

### 4.7 Seahorse Analysis

The Seahorse XFe24 cell metabolism analyzer (XFe24, Agilent Technologies) was employed to assess cellular metabolic activity. To determine the oxygen consumption rate (OCR), isolated splenic B cells (2 × 10^6^) were first activated with F(ab')_2_ anti‐mouse Ig (M + G) for 2 h. These cells were then treated with a series of metabolic modulators in sequence: 1.5 µM oligomycin, 1 µM FCCP, 500 nM rotenone, and 1 µM antimycin A, all prepared in a poly‐D‐lysine solution (50 µg/mL, C0312, Beyotime) within a precoated 24‐well plate. For the extracellular acidification rate (ECAR) measurement, cells were initially incubated overnight with F(ab')_2_ Ig (M + G) and subsequently exposed to 10 mM glucose, 2 µM oligomycin, and 5 mM 2‐deoxyglucose (2‐DG) in a stepwise manner.

### 4.8 Transmission Electron Microscopy

B cells (1 × 10^6^) were isolated from WT and KO mice. Initial fixation was performed with 2.5% glutaraldehyde for 2 h at 4°C. Postfixation and staining involved treatment with 2% OsO4 in cacodylate buffer for 1 h at 4°C. Subsequently, cells underwent gradual dehydration through an ascending ethanol series and were then embedded in Epon resin. Ultrathin sections, prepared using an ultramicrotome, were mounted onto Formvar‐coated slot grids and contrasted with lead citrate. Images were acquired using a Hitachi H‐7000FA TEM (Japan).

### 4.9 Scanning Electron Microscopy

To visualize B cell morphology by SEM, poly‐d‐lysine‐treated sterile coverslips were first incubated for 3 h at 37°C with 10 µg/mL biotinylated F(ab')_2_ Ig (M+G). Onto these prepared surfaces, 2 × 10^6^ purified B cells from WT and KO mice were added and allowed to stimulate for 10 min at 37°C. After washing, cells were preserved with 2.5% glutaraldehyde. Dehydration was carried out with a series of increasing ethanol concentrations, followed by drying to the critical point with liquid carbon dioxide. An SU8010 ultra‐high‐resolution SEM (Hitachi) captured the images. Analysis of filopodial expansion was performed with ImageJ (Bethesda).

### 4.10 ELISA

The expression levels of IgA and Fgl2 in plasma were detected with kits for IgA (cat. no. E99‐103, Mouse IgA ELISA Kit) and Fgl2 (MOFI00808, Mouse Fgl2 [fibrinogen‐like protein 2] ELISA kit).

### 4.11 Single‐Cell Sequencing and Single‐Cell BCR Sequencing

Single‐cell RNA‐seq libraries were constructed following the manufacturer's protocol using the 10× Genomics Single Cell 3′ Reagent Kit v2. Approximately 22,000 cells at 2000 cells/µL were encapsulated into droplets, lysed, and subjected to reverse transcription (53°C, 45 min; 85°C, 5 min) in a Thermo Fisher 96‐well thermal cycler (Cat# 4375786). After droplet disruption, barcoded cDNA was purified using DynaBeads (Cat# 37002D) and amplified by PCR (98°C, 3 min; 14 cycles of 98°C/15 s, 67°C/20 s, 72°C/1 min; 72°C, 1 min). The cDNA was then fragmented, adapter‐ligated, size‐selected (∼300 bp) with SPRI beads (Cat# B23318), and finally sequenced on an Illumina HiSeq XTEN platform.

### 4.12 Statistical Analysis

Normality of all data was confirmed before statistical evaluation. GraphPad Prism (8.0.1) was utilized for conducting two‐tailed unpaired Student's *t*‐tests. Flow cytometry data concerning bone marrow or peripheral B‐cell populations were normalized based on the total cellularity of the corresponding tissue for statistical treatment. The degree of colocalization was determined using Pearson's correlation coefficient. Findings are derived from a minimum of three separate experiments, and the displayed images are illustrative examples. Error bars depict the mean and standard deviation (SD). Statistical significance was accepted for *p* < 0.05.

## Author Contributions

**Jiang Chang**: formal analysis, investigation, methodology, project administration, validation, visualization, writing – original draft. **Da Huang**: formal analysis, investigation. **Wei Yuan**: investigation, methodology. **Jianing Tang**: investigation, resources. **Jingzhi Yang**: investigation, resources. **Yuying Chen, Zhize Yuan, and Yizhi Wu**: resources. **Di Wu**: conceptualization, funding acquisition. **Weiming Yan**: conceptualization, supervision, writing – original draft. **Qin Ning**: conceptualization, funding acquisition, writing – review and editing. All authors have read and approved the final manuscript.

## Funding

This work was supported by grants from the National Key Research and Development Program of China (2023YFC2308600, 2021YFC2600200) and the National Natural Science Foundation of China (82202502, 82402606, 82400706).

## Ethics Statement

The authors have nothing to report.

## Conflicts of Interest

Shanghai Honest Biotechnology, Ltd. conducted the proteomics and metabolomics assays and provided support for bioinformatic analysis. These services did not affect the study's design, results, or interpretation. The authors declare no conflicts of interest.

## Supporting information




**Figure S1** (a) Immunofluorescence analysis was performed on the spleen sections of WT mice and WT mice infected with *T.s*. for Fgl2 and IgA (Fgl2 was labeled in red, and IgA in green). Representative images were acquired with a 60× objective. Scale bar, 10 µm. (b, c) Detection of Fgl2 and IgA expression levels in plasma by ELISA in WT mice and WT mice infected with *T.s*. (WT *n* = 5, KO *n* = 5). (d–g) Flow cytometric analysis of T follicular helper (Tfh; CD4+TCR‐β+CXCR5+PD‐1+) and regulatory B cells (Bregs; CD19+IL‐10+) in the spleen, mLNs, and PPs of *T. spiralis*‐infected WT and Fgl2‐KO mice (*n* = 5 per group). Representative plots and summary graphs of the percentages of these subsets are shown (WT *n* = 5, KO *n* = 5). (H) Quantification of *Trichinella* larvae recovered from the duodenum of infected WT and KO mice. Error bars represent the mean (±SD). *p < 0.05, **p < 0.01, ****p < 0.0001, ns: no significant difference.
**Figure S2** (a) Representative flow cytometry plots showing the percentages of IgG1+, IgG2b+, and IgE+ B cells in the spleen, mLNs, and PPs of *T. spiralis*‐infected WT and KO mice (*n* = 5 per group). (b–h) Flow cytometric analysis of splenic B cell subsets from WT and KO mice at the muscle stage of *T. spiralis* infection (5 weeks postinfection). Representative plots and summary graphs show the percentages of FO B cells, GC B cells, plasmablasts, plasma cells, IgA+ plasma cells, and class‐switched B cells (IgG1+, IgG2b+, IgA+, IgE+). Muscle Trichinella infection stage: After the intestinal Trichinella infection, mice were infected for more than 5 weeks, and euthanized by cervical dislocation. Spleen and all muscle tissues were collected. (j) Quantification of *Trichinella* larvae recovered from the muscle tissue of WT and KO mice at the muscle stage of infection (*n* = 5 per group). Error bars represent the mean (±SD). *p < 0.05, ***p < 0.001, ns: no significant difference.
**Figure S3** (a) Diagram defining genetic markers of various cell subpopulations. (b) UMAP plot showing highly expressed genes in B‐cell_sp1, B‐cell_sp2, B‐cell_sp3, and B‐cell_sp4. (c) Expression levels of *Pdcd1, Havcr2, Ctla4*, and *Icos* genes in WT and KO B cells. (d) Single‐cell transcriptomics analysis of Fgl2 expression levels across different B cell subsets.
**Figure S4** (a) Schematic of the bone marrow (BM) transplantation experiment. BM cells from WT or KO (CD45.2) mice were mixed with BM cells from WT (CD45.1) mice at a 1:1 ratio. Recipient WT mice (CD45.1) were preirradiated with 7 Gy X‐rays and intravenously injected with 5 × 10^6^ mixed cells. Eight weeks after bone marrow chimerism, recipient mice's spleen B cells were analyzed. (b–e) Representative flow cytometric plots of B cell subsets in CD45.2 WT and KO chimeras. (f) Quantitative analysis of the proportion of FO B, MZ B, GC B, T1, and T2 cells in the CD45.2 population (WT mice, *n* = 5; KO mice, *n* = 5). (g) Quantitative analysis of the proportion of FO B, MZ B, GC B, T1, and T2 cells in the CD45.1 population (WT mice, *n* = 5; KO mice, *n* = 5). (h) Quantitative analysis of the proportion of FO B, MZ B, GC B, T1, and T2 cells in the CD45.1 population of WT mice and the CD45.2 population of KO mice (WT mice, *n* = 5; Fgl2 KO mice, *n* = 5). Data are presented as mean ± SD. *p < 0.05, ***p < 0.001, ns: not significant.
**Figure S5** (a) Representative flow cytometry plots showing the adhesion molecules and integrin markers α4, β7, αL, β2, CXCR4, CCR7, and CD62L on blood B cells from WT and KO mice. (b, c) Representative dot plots of B cell precursors in the BM. The boxes show the proportion of each B cell precursor in the total BM cell population: pre‐pro B cells (a), pro‐B cells (b), early pre‐B cells (c), late pre‐B cells (d), immature B cells (e), and recirculating mature B cells (f). (d) Quantitative analysis of the percentage and absolute number of B cell precursors in the BM (*n* = 9). (e) Mean fluorescence intensity (MFI) of CD127 in different B cell subsets. (f) Representative dot plots showing B1a (CD19^+^IgD^−^IgM^+^CD5^+^CD11b^+^) and B1b (CD19^+^IgD^−^IgM^+^CD5^−^CD11b^+^) cells in the peritoneal cavity. (g, h) Quantitative analysis of the percentage and absolute number of B1a and B1b cells in the peritoneal cavity (WT: *n* = 5, KO: *n* = 5). *p < 0.05, **p < 0.01, ***p < 0.001, ****p < 0.0001, ns: no statistical significance.
**Figure S6** (a–c) Stimulation of splenic B cells from WT and KO mice with AF594 F(ab')_2_ goat anti‐mouse IgG + IgM (10 µg/mL) for 0 and 5 min, followed by fixation, permeabilization, and staining for F‐actin and pWASP. Pearson correlation analysis was performed to examine the co‐localization of F‐actin and pWASP with the BCR (confocal microscopy). (d) Electron microscopy images showing the extension of filopodia in activated B cells (scale bar = 5 µm). (e) Quantification of filopodia number and length in activated B cells. (f, g) Spreading area of B cells activated with anti‐CD40 + IL‐4. Wide‐field fluorescence microscopy images of B cells stained for F‐actin (green), BCR (red), and DAPI (Hoechst 33258, blue) (The detailed experimental procedures were conducted following established protocols ^1^). (h) Representative total internal reflection fluorescence microscopy (TIRFm) images of pSYK activation at 3, 5, and 7 min (100× objective, scale bar = 2.5 µm). (i) Representative TIRFm images of pSHIP‐1 activation at 3, 5, and 7 min (100× objective, scale bar = 2.5 µm). (j) Quantification of the B cell contact area (under interference reflection microscopy) and the mean fluorescence intensity (MFI) of BCR, pSYK, and pSHIP‐1 (confocal data). Data were measured by using NIS‐Elements AR 3.2 software. Scale bars = 2.5 µm. *p < 0.05, **p < 0.01, ***p < 0.001, ****p < 0.0001, ns: no statistical significance, Mann–Whitney *U*‐test.
**Table S1**: Sample information of the clinical cohort of patients in the early stage of COVID‐19.
**Table S2**: Antibodies used in the study.

## Data Availability

All the data are included in the manuscript and the additional file. All the materials and reagent sources used in this study are described in the Materials and Methods section. The mass spectrometry proteomics data have been deposited in the ProteomeXchange Consortium (https://proteomecentral.proteomexchange.org) via the iProX partner repository under dataset identifier PXD055099. The data can be accessed at http://proteomecentral.proteomexchange.org/cgi/GetDataset?ID=PXD055099. Our scRNA‐seq and scBCR‐seq data have been uploaded to the GEO database under accession number GSE275827.
