# Supplementary material for: Fibrinogen‐Like Protein 2 Modulates B Cell Mucosal Immunity by Suppressing Receptor for Activated C‐Kinase 1‐Mediated AKT Phosphorylation
Source: MedComm (2020). 2026 Mar 24;7(4):e70633. doi: 10.1002/mco2.70633 (PMC13042553; doi:10.1002/mco2.70633)

**Supplementary Materials for**

**Fibrinogen-like protein 2 Modulates B Cell Mucosal Immunity by Suppressing Receptor for activated C-kinase 1-Mediated AKT Phosphorylation**

**^1^Jiang Chang, ^1^Da Huang, ^1^Wei Yuan, ^1^Jianing Tang, ^2^Jingzhi Yang, ^1^Yuying Chen, ^1^Zhize Yuan, ^1^Yizhi Wu, ^1*^Di Wu, ^1*^Weiming Yan, ^1*^Qin Ning**

^1^Department and Institute of Infectious Disease, Tongji Hospital, Tongji Medical College and State Key Laboratory for Diagnosis and Treatment of Severe Zoonotic Infectious Diseases, Huazhong University of Science and Technology, Wuhan 430030, Hubei Province, China.

^2^Department of Orthopedics, Qilu Hospital of Shandong University, Jinan, Shandong 250063, PR China

***Corresponding author**

Di Wu: [woody_1984@163.com](mailto:woody_1984@163.com)

Weiming Yan: ywm_net@tjh.tjmu.edu.cn

Qin Ning: qning@vip.sina.com

**Supplementary Figures and legends:**


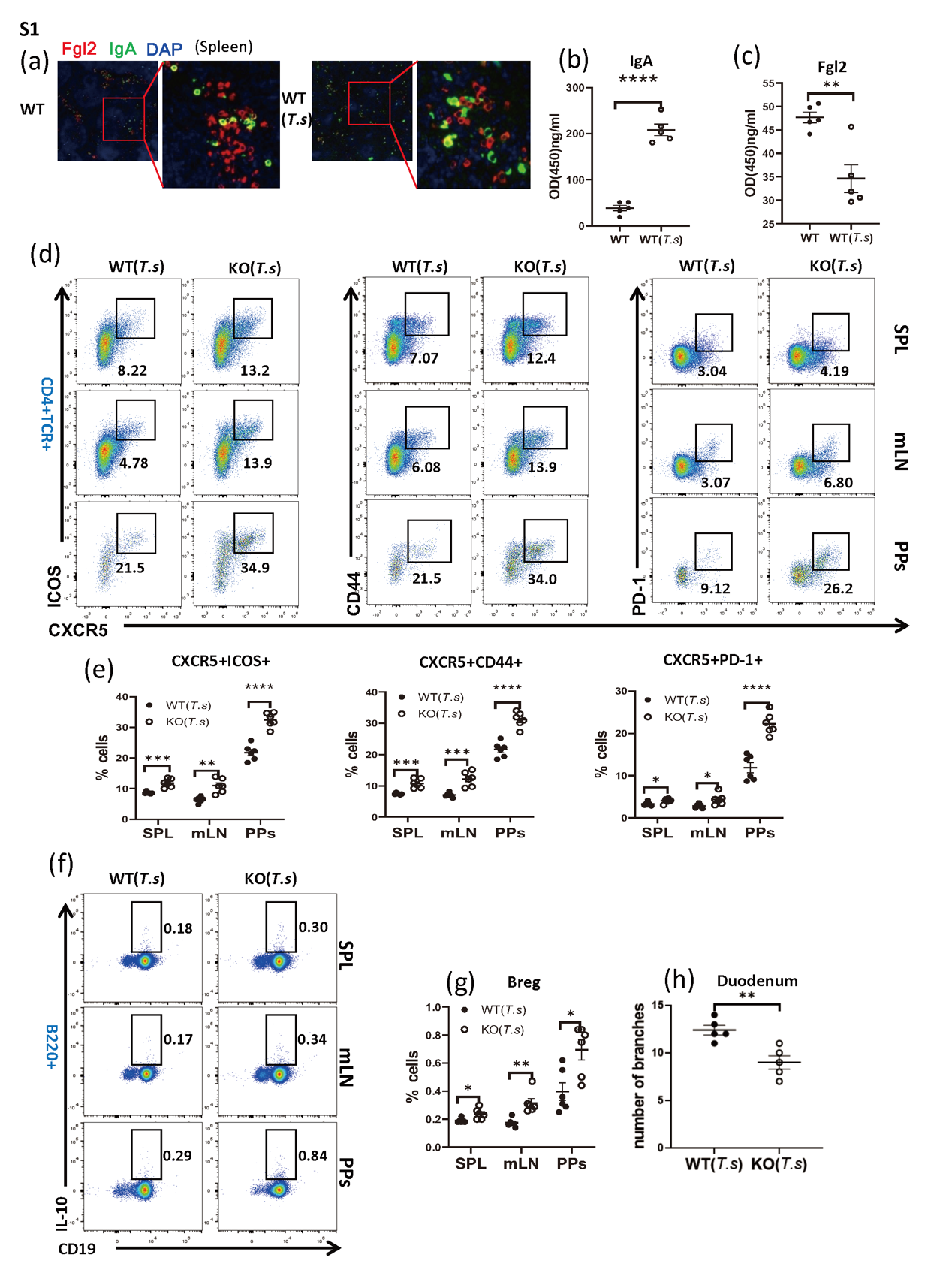


Fig S1 (a) Immunofluorescence analysis was performed on the spleen sections of WT mice and WT mice infected with *T.s*. for Fgl2 and IgA (Fgl2 was labeled in red, and IgA in green). Representative images were acquired with a 60x objective. Scale bar, 10 μm.

(b, c) Detection of Fgl2 and IgA expression levels in plasma by ELISA in WT mice and WT mice infected with *T.s*. (WT n = 5, KO n = 5)

(d-g) Flow cytometric analysis of T follicular helper (Tfh; CD4+TCR-β+CXCR5+PD-1+) and regulatory B cells (Bregs; CD19+IL-10+) in the spleen, mLNs, and PPs of *T. spiralis*-infected WT and Fgl2-KO mice (n=5 per group). Representative plots and summary graphs of the percentages of these subsets are shown (WT n = 5, KO n = 5).

(h) Quantification of *Trichinella* larvae recovered from the duodenum of infected WT and KO mice.

Error bars represent the mean (± SD). *p < 0.05, **p < 0.01, ****p < 0.0001, ns: no significant difference.


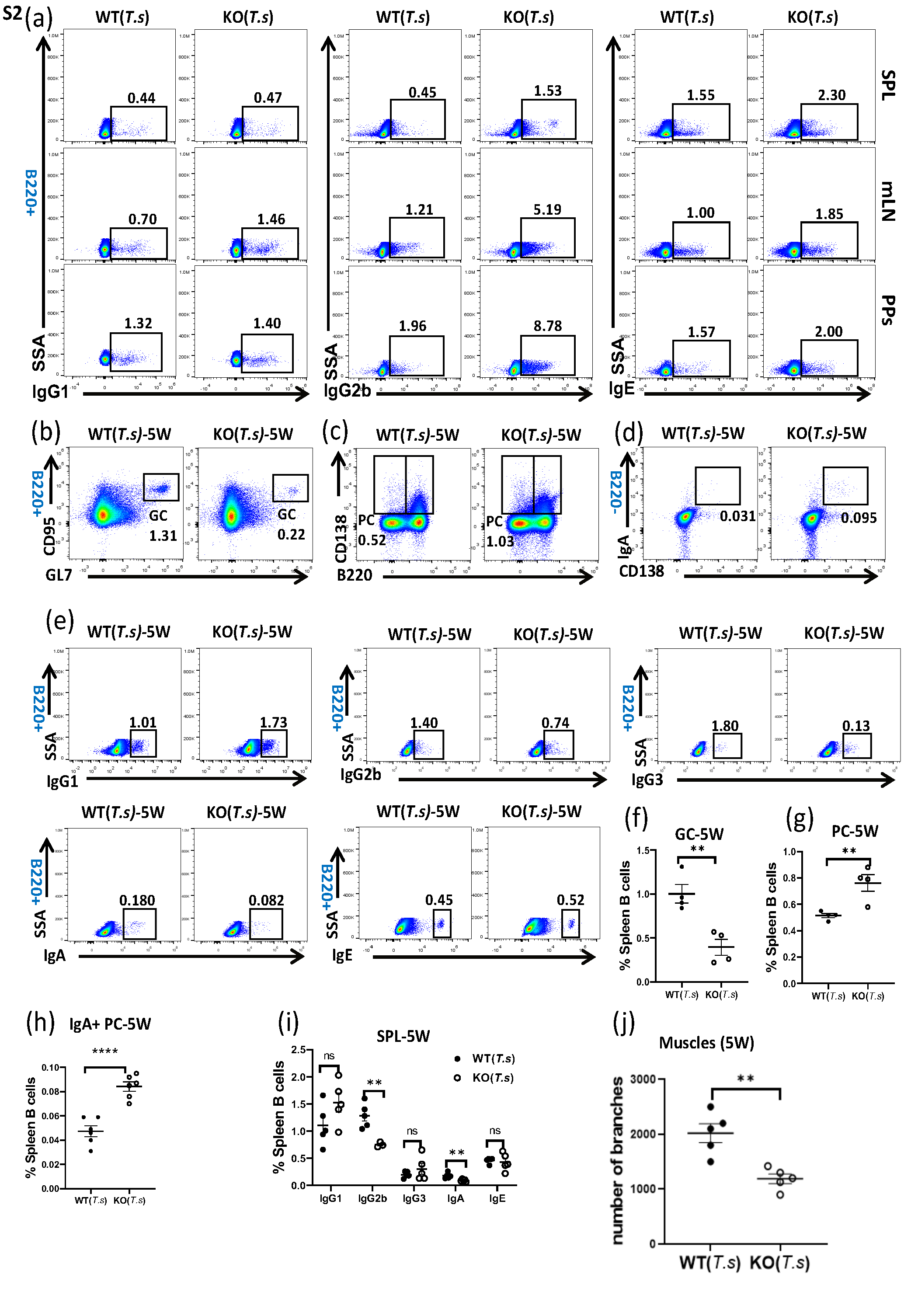


Fig S2 (a) Representative flow cytometry plots showing the percentages of IgG1+, IgG2b+, and IgE+ B cells in the spleen, mLNs, and PPs of *T. spiralis*-infected WT and KO mice (n=5 per group).

(b-h) Flow cytometric analysis of splenic B cell subsets from WT and KO mice at the muscle stage of *T. spiralis* infection (5 weeks post-infection). Representative plots and summary graphs show the percentages of FO B cells, GC B cells, plasmablasts, plasma cells, IgA+ plasma cells, and class-switched B cells (IgG1+, IgG2b+, IgA+, IgE+). Muscle Trichinella Infection Stage: after the intestinal Trichinella infection, mice were infected for more than 5 weeks, and euthanized by cervical dislocation. Spleen and all muscle tissues were collected.

(j) Quantification of *Trichinella* larvae recovered from the muscle tissue of WT and KO mice at the muscle stage of infection (n=5 per group).

Error bars represent the mean (± SD). *p < 0.05, ***p < 0.001, ns: no significant difference.


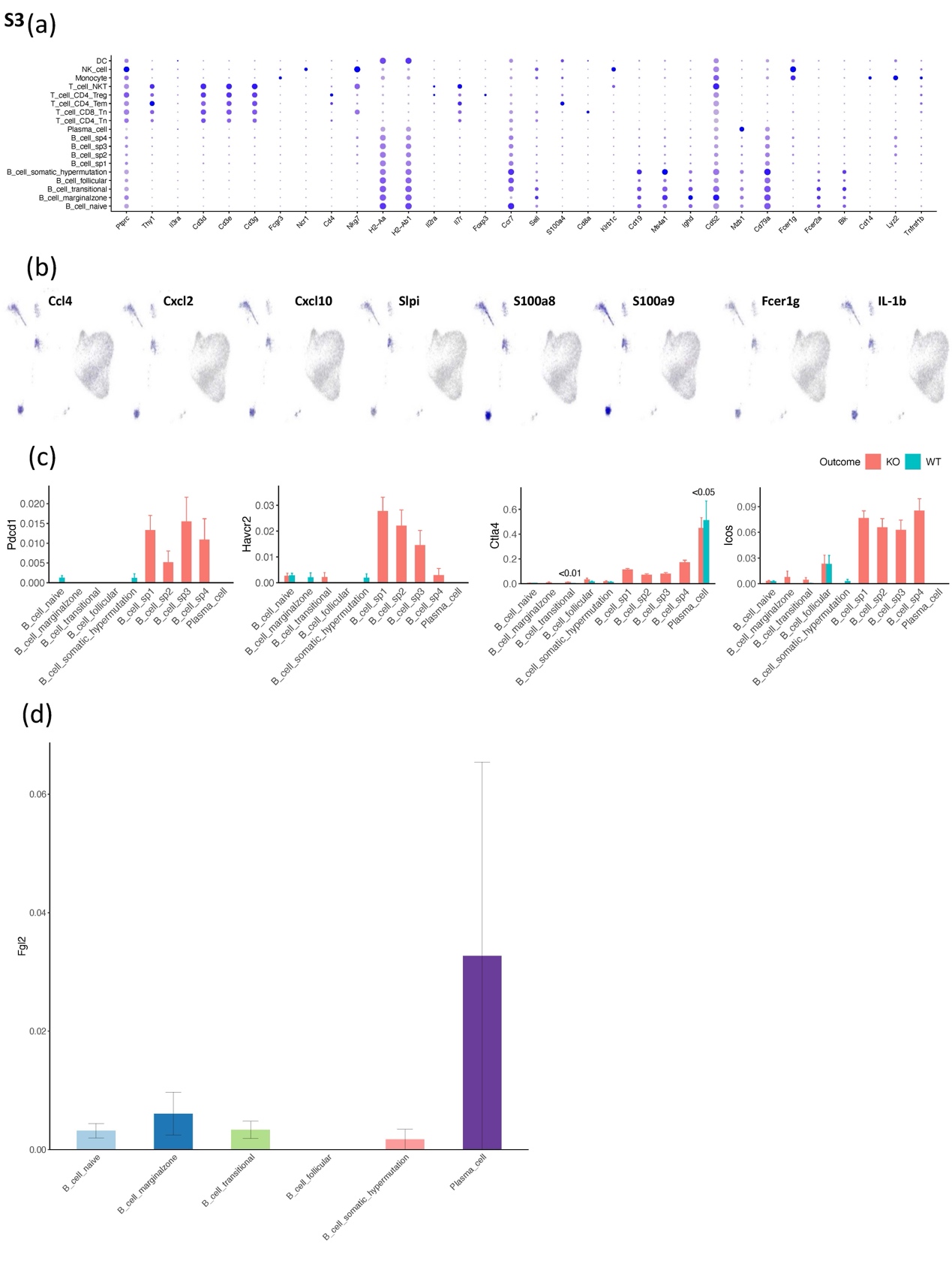


Fig S3 (a) Diagram defining genetic markers of various cell subpopulations.

(b) UMAP plot showing highly expressed genes in B-cell_sp1, B-cell_sp2, B-cell_sp3 and B-cell_sp4.

(c) Expression levels of *Pdcd1, Havcr2, Ctla4* and *Icos* genes in WT and KO B cells.

(d) Single-cell transcriptomics analysis of Fgl2 expression levels across different B cell subsets.
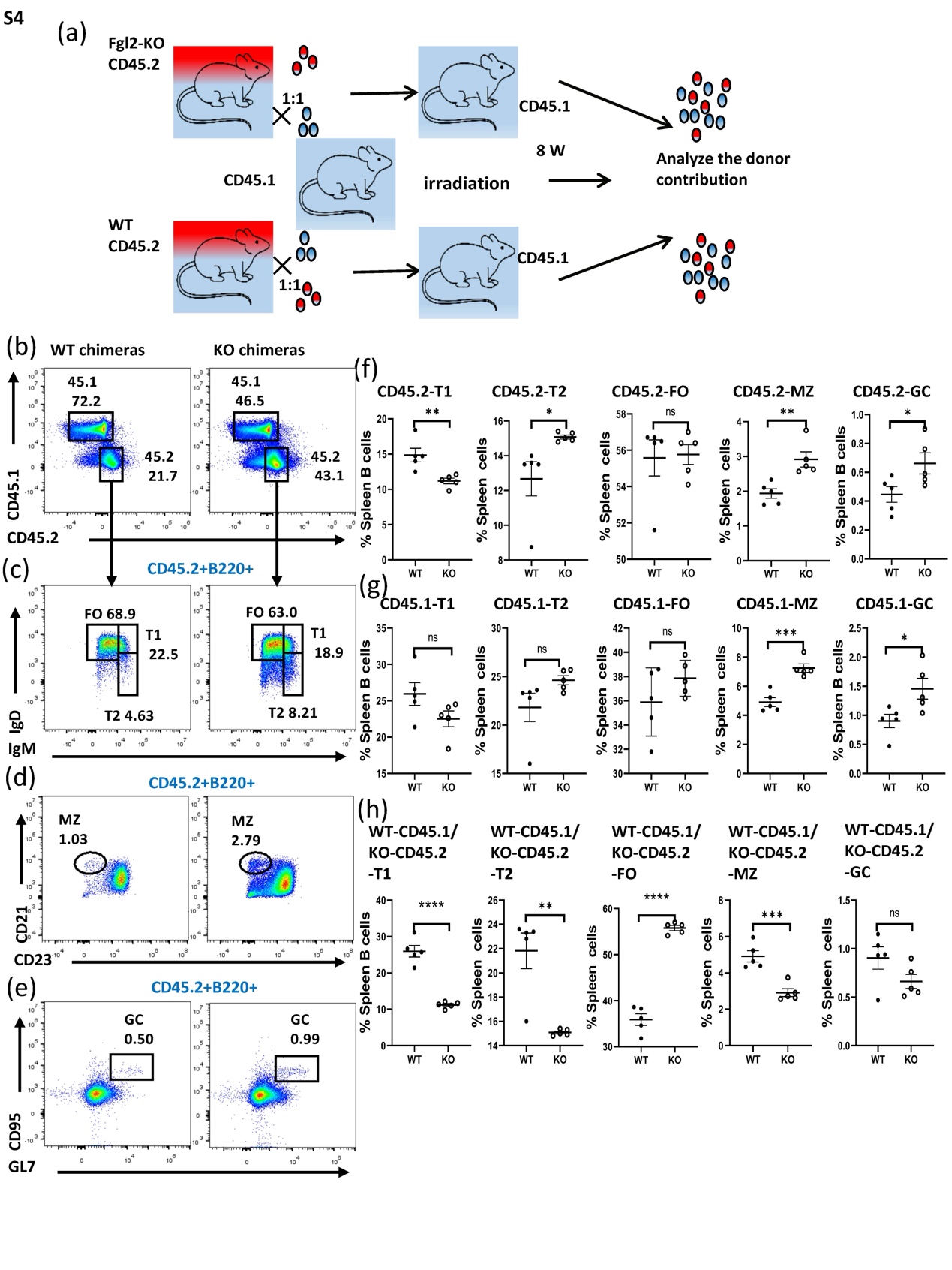


Fig S4 (a) Schematic of the bone marrow (BM) transplantation experiment. BM cells from WT or KO (CD45.2) mice were mixed with BM cells from WT (CD45.1) mice at a 1:1 ratio. Recipient WT mice (CD45.1) were pre-irradiated with 7 Gy X-rays and intravenously injected with 5 × 10⁶ mixed cells. Eight weeks after bone marrow chimerism, recipient mice spleen B cells were analyzed.

(b-e) Representative flow cytometric plots of B cell subsets in CD45.2 WT and KO chimeras.

(f) Quantitative analysis of the proportion of FO B, MZ B, GC B, T1, and T2 cells in the CD45.2 population (WT mice, n=5; KO mice, n=5).

(g) Quantitative analysis of the proportion of FO B, MZ B, GC B, T1, and T2 cells in the CD45.1 population (WT mice, n=5; KO mice, n=5).

(h) Quantitative analysis of the proportion of FO B, MZ B, GC B, T1, and T2 cells in the CD45.1 population of WT mice and CD45.2 population of KO mice (WT mice, n=5; Fgl2 KO mice, n=5).

Data are presented as mean ± SD. *p < 0.05, ***p < 0.001, ns: not significant.


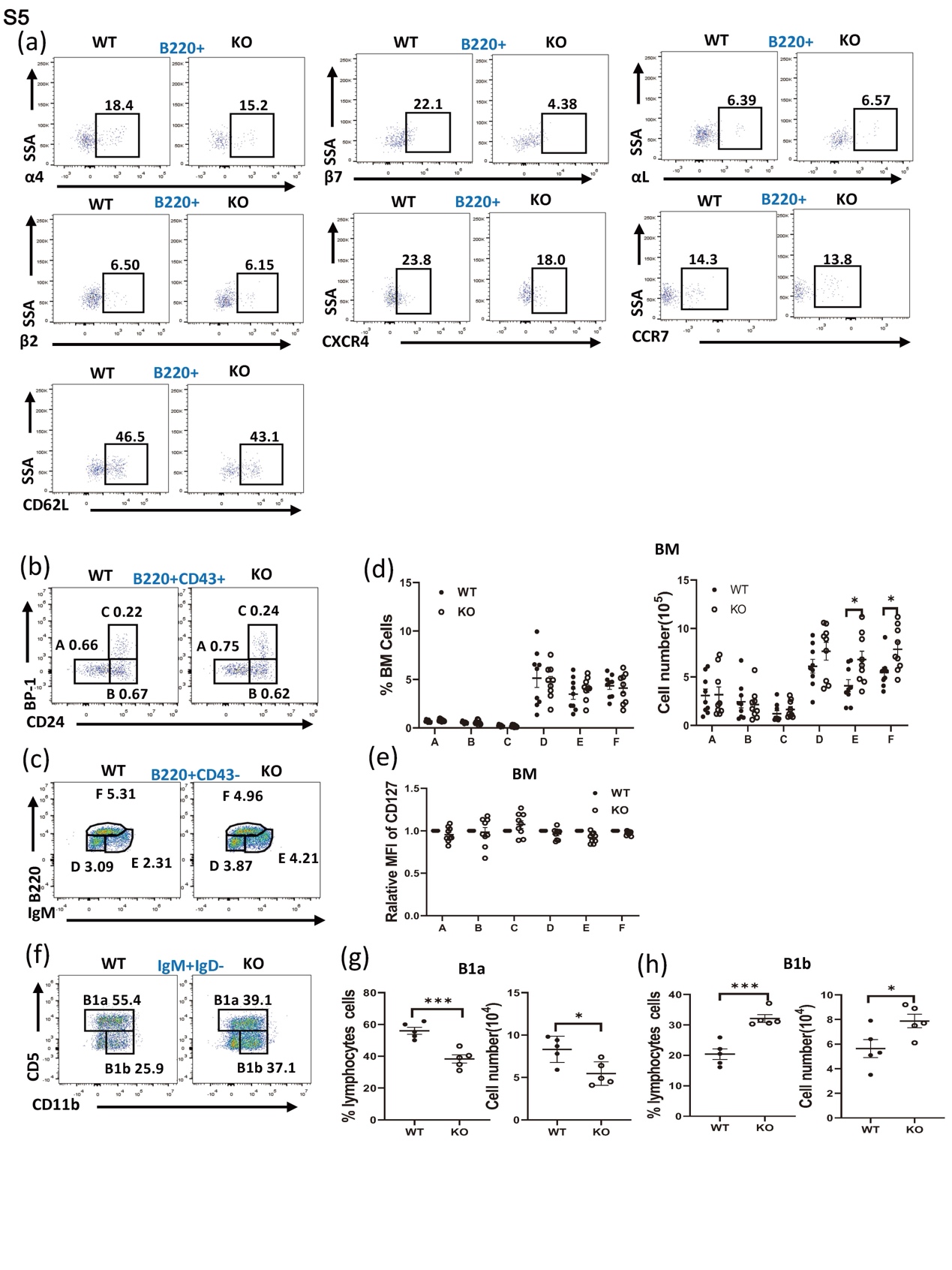


Fig S5 (a) Representative flow cytometry plots showing the adhesion molecules and integrin markers α4, β7, αL, β2, CXCR4, CCR7, and CD62L on blood B cells from WT and KO mice.

(b, c) Representative dot plots of B cell precursors in the BM. The boxes show the proportion of each B cell precursor in the total BM cell population: Pre-pro B cells (A), Pro B cells (B), Early Pre-B cells (C), Late Pre-B cells (D), Immature B cells (E), and Recirculating Mature B cells (F).

(d) Quantitative analysis of the percentage and absolute number of B cell precursors in the BM (n = 9).

(e) Mean fluorescence intensity (MFI) of CD127 in different B cell subsets.

(f) Representative dot plots showing B1a (CD19+IgD⁻IgM+CD5+CD11b+) and B1b (CD19+IgD⁻IgM+CD5⁻CD11b+) cells in the peritoneal cavity.

(g, h) Quantitative analysis of the percentage and absolute number of B1a and B1b cells in the peritoneal cavity (WT: n = 5, KO: n = 5). *p < 0.05, **p < 0.01, ***p < 0.001, ****p < 0.0001, ns: no statistical significance.


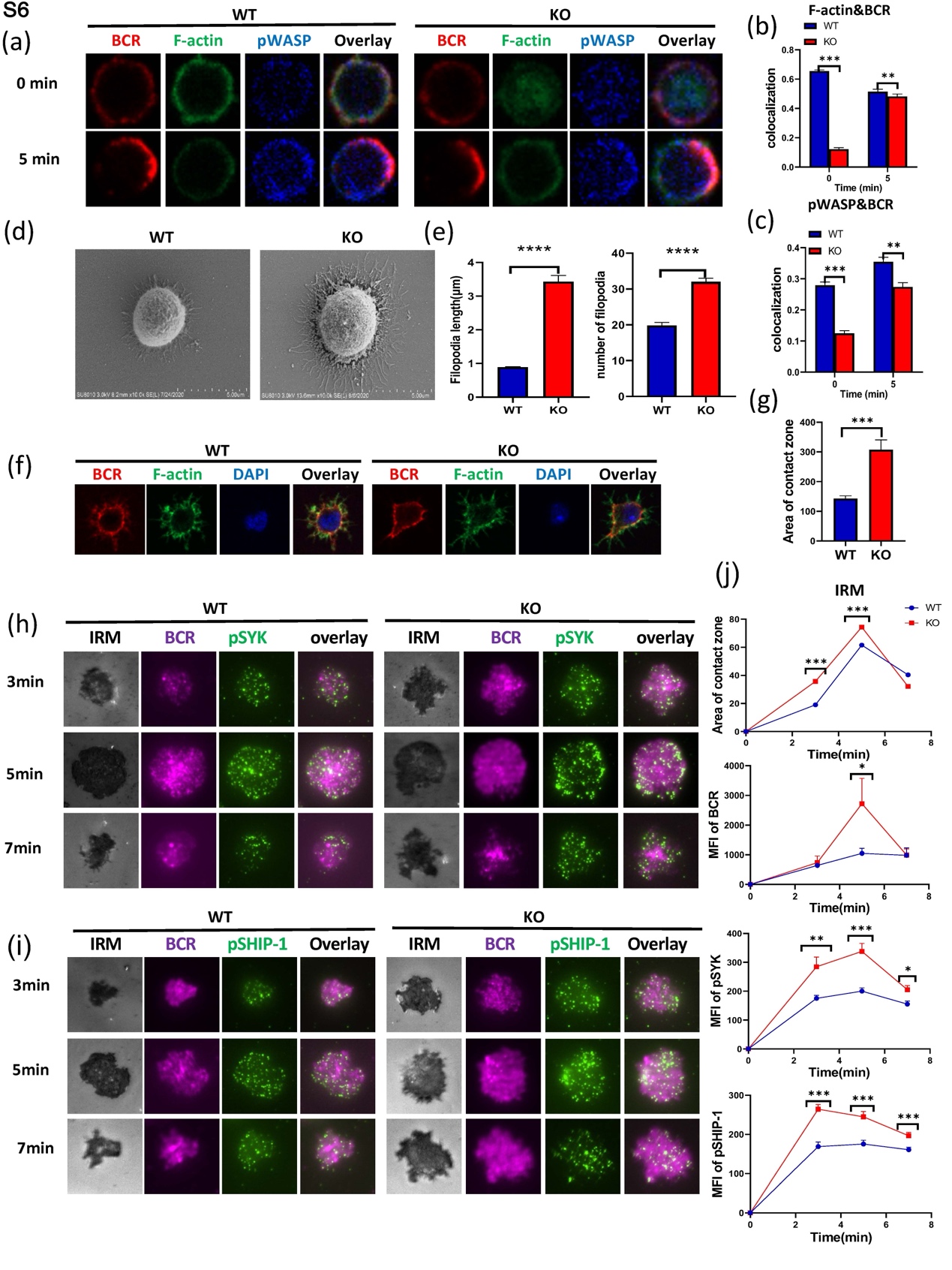


Fig S6 (a-c) Stimulation of splenic B cells from WT and KO mice with AF594 F(ab')_2_ goat anti-mouse IgG + IgM (10 μg/ml) for 0 and 5 min, followed by fixation, permeabilization, and staining for F-actin and pWASP. Pearson correlation analysis was performed to examine the co-localization of F-actin and pWASP with the BCR (confocal microscopy).

(d) Electron microscopy images showing the extension of filopodia in activated B cells (scale bar = 5 μm).

(e) Quantification of filopodia number and length in activated B cells.

(f, g) Spreading area of B cells activated with anti-CD40 + IL-4. Wide-field fluorescence microscopy images of B cells stained for F-actin (green), BCR (red), and DAPI (Hoechst 33258, blue) (The detailed experimental procedures were conducted following established protocols ^1^ ).

(h) Representative total internal reflection fluorescence microscopy (TIRFm) images of pSYK activation at 3, 5, and 7 minutes (100× objective, scale bar = 2.5 μm).

(i) Representative TIRFm images of pSHIP-1 activation at 3, 5, and 7 minutes (100× objective, scale bar = 2.5 μm).

(j) Quantification of the B cell contact area (under interference reflection microscopy) and the mean fluorescence intensity (MFI) of BCR, pSYK, and pSHIP-1 (confocal data).

Data were measured by using NIS-Elements AR 3.2 software. Scale bars = 2.5 μm. *p < 0.05, **p < 0.01, ***p < 0.001, ****p < 0.0001, ns: no statistical significance, Mann-Whitney U test.

**Reference**

1. Gerasimcik N, Westerberg LS, Severinson E. Methods to Study the Role of Cdc42, Rac1, and Rac2 in B-Cell Cytoskeletal Responses. *Methods Mol Biol*. 2018;1821:235-246.

**Table S1**

This study included eight young patients with laboratory-confirmed COVID-19 from the fever clinic of Tongji Hospital who presented with upper respiratory tract symptoms. Peripheral blood mononuclear cells (PBMCs) were isolated from peripheral blood samples collected from these patients. The clinical characteristics of these patients are summarized in Supplementary Table 1.

| ID | Age (years) | Sex | COVID-19（+/-） | Upper respiratory tract symptoms | duration from symptom onset to sampling |
| --- | --- | --- | --- | --- | --- |
| 1 | 26 | Male | + | Yes | <48h |
| 2 | 25 | Male | + | Yes | <48h |
| 3 | 28 | Male | + | Yes | <48h |
| 4 | 27 | Male | + | Yes | <48h |
| 5 | 29 | Male | + | Yes | <48h |
| 6 | 27 | Male | + | Yes | <48h |
| 7 | 25 | Male | + | Yes | <48h |
| 8 | 30 | Male | + | Yes | <48h |

**Table S1: Sample information of the clinical cohort of patients in the early stage of COVID-19.**

**Table S2**

| Antibody | Supplier | Reference |
| --- | --- | --- |
| FITC–anti-CD127 | BioLegend | 135008 |
| APC–anti-CD43 | BioLegend | 143208 |
| PE–anti–BP-1 | BioLegend | 108307 |
| FITC–anti-CD19 | BioLegend | 101506 |
| PE–anti-CD23 | BioLegend | 101608 |
| APC–anti-CD21 | BioLegend | 123412 |
| PerCP/Cy5.5–anti-IgD | BioLegend | 405710 |
| BV510–anti-B220 | BioLegend | 103247 |
| BV421–anti- IgM | BioLegend | 406518 |
| Alexa Fluor 647 (AF647)–anti-GL7 | BioLegend | 144606 |
| FITC–anti-B220 | BioLegend | 103206 |
| PE-Cy7-anti-CD5 | BioLegend | 100622 |
| APC-Cy7-anti-CD11b | BioLegend | 101226 |
| BV510-anti-CD138 | BioLegend | 142521 |
| APC/Cy7–anti-CD45.1 | Biolegend | 110716 |
| BV510–anti-CD45.2 | Biolegend | 109838 |
| FITC anti-CD29 | Biolegend | 102205 |
| FITC anti-CD11a | Biolegend | 101106 |
| FITC anti-CD49d | Biolegend | 103606 |
| PE anti-CD18 | Biolegend | 101407 |
| APC anti-β7 | Biolegend | 321208 |
| PE anti-CD184 | Biolegend | 146506 |
| APC anti-CCR7 | Biolegend | 120108 |
| PE-CY7- anti-CD62L | Biolegend | 104418 |
| APC/Cy7–anti-CD45.1 | Biolegend | 110716 |
| BV510–anti-CD45.2 | Biolegend | 109838 |
| APC-anti-CD4 | BioLegend | 100 516 |
| PE-Cy7-anti-PD-1 | BioLegend | 109 110 |
| APC-anti-IL-10 | BioLegend | 505009 |
| FITC-anti-IgA | BD Biosciences | 559 354 |
| FITC-anti-IgG3 | BD Biosciences | 553 403 |
| PE-anti-IgG1 | BD Biosciences | 550 083 |
| FITC-anti-IgE | BD Biosciences | 553 415 |
| FITC-anti-IgG2b | BD Biosciences | 553 395 |
| Rat-anti-mouse CXCR5 | BD Biosciences | 551 961 |
| PE-Cy5-anti-CD19 | BD Biosciences | 555414 |
| PE-Cy7-anti-CD38 | BD Biosciences | 560677 |
| BV605-anti-CD24 | BD Biosciences | 563788 |
| PE-anti-CD27 | BD Biosciences | 555441 |
| Anti-Fgl2- antibody | abnova | H00010875-M01 |
| Anti- Rack1 antibody | Santa | sc-17754 |
| Anti-IgG Light Chain antibody | Abbkine | A25012 |
| Anti-pBTK antibody | Abcam | ab52192 |
| Anti-pWASP antibody | Bethyl | A300-205A |
| Anti-pAKT antibody | Cell Signaling Technology | 9272S |
| Anti-pSHIP antibody | Cell Signaling Technology | 3941S |
| Anti-pPI3K antibody | Cell Signaling Technology | 4228S |
| Anti-pFOXO1 antibody | Cell Signaling Technology | 9461S |
| Anti-pS6 antibody | Cell Signaling Technology | 4856S |
| Anti-pmTOR antibody | Cell Signaling Technology | 5536S |
| Anti-pSYK antibody | Cell Signaling Technology | 2710S |
| Anti-DOCK8 antibody | Cell Signaling Technology | 39263S |
| Anti-pY antibody | merck-millipore | 05-321 |
| Biotin- F(ab')^2^ anti-human Ig (M + G) | Jackson ImmunoResearch | 109-066-127 |
| Biotin-conjugated F(ab')^2^ anti-mouse Ig (M + G) | Jackson ImmunoResearch | 115-066-068 |
| Alexa Fluor 488 goat anti-mouse IgG | Jackson ImmunoResearch | 715-165-151 |
| Anti-pY antibody | Merck-Millipore | 05-321 |
| Anti- β-actin antibody | Proteintech | 60008-1-IG-10 |
| Anti-IgG antibody | SantaCruz | sc-2025 |
| HRP conjugated Goat anti-Rabbit IgG (H+L) | Servicebio | GB23303 |
| Alexa Fluor 488 goat anti-rabbit IgG | Thermo Fisher | A-11008 |
| Alexa Fluor 405 goat anti-rabbit IgG | Thermo Fisher | A-31556 |
| Alexa Fluor 647 goat anti-rabbit IgG | Thermo Fisher | A-21245 |
| Alexa Fluor 594 F(ab')^2^ goat anti mouse IgM+IgG (H+L) | Jackson ImmunoResearch | 115-586  068 |

**Table S2: Antibodies used in the study.**

**Supplementary Information of methodological descriptions**

**Yeast Two-hybrid System**

Total RNA was extracted from splenic B cells of wild-type mice to construct a cDNA library by recombining cDNA into the pGADT7-Rec vector using Exnase™ II. The library quality was evaluated via transformation efficiency assessment and colony PCR to determine the average insert length. For screening, the *Fgl2* gene was cloned into the pGBKT7 bait vector and screened against the constructed library using the Y2H system (Takara, Japan). The identified candidate, *Rack1*, was cloned into the pGADT7 vector for verification. Interactions were confirmed by one-to-one validation on DDO, TDO, and QDO selective plates after excluding self-activation and toxicity in the Y2H Gold strain.

**Bone Marrow Chimeras**

Two-to-three-month-old B6 mice (CD45.1) were irradiated with 7 Gy X-rays and immediately injected intravenously with 5 × 10^6^ bone marrow cells from WT or *Fgl2*-KO (CD45.2) mice under aseptic conditions. Cells from WT or *Fgl2*-KO mice were mixed with those from CD45.1 mice at a 1:1 ratio. Water with antibiotics (Gentamicin 480,000 U/L and erythromycin 375,000 U/L) was administered to the mice 1 week before radiation and 2 weeks after the injection. Eight weeks after transfer, the recipient mice were sacrificed and analyzed by flow cytometry.

***T. spiralis* Infection Model**

Mice were infected with *T. spiralis* for more than 45 days and sacrificed by neck dissection. All muscles were separated, and the larvae were cut, digested, and collected for counting. Subsequently, the larvae were intragastrically injected into WT (*T. spiralis*) and KO mice (*T. spiralis*) at the density of 200–400 larva per 100–200 μl system. After 5–7 days of infection, the mice were euthanized, and the abdominal cavity was opened. The small intestine was removed, and the adult worms were collected and counted.

**The original Western Blot images:**


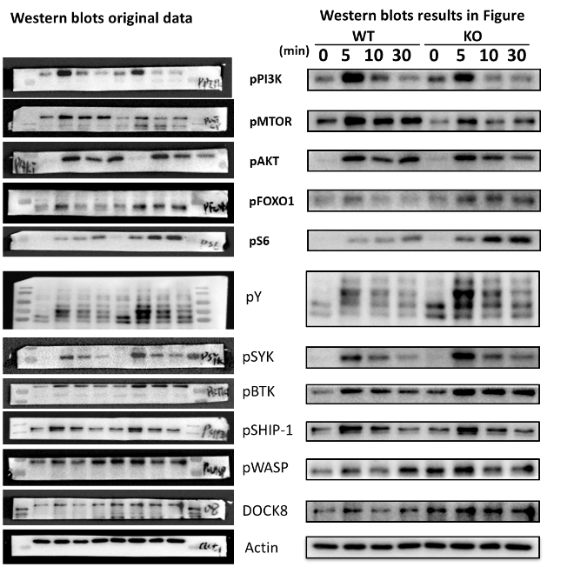


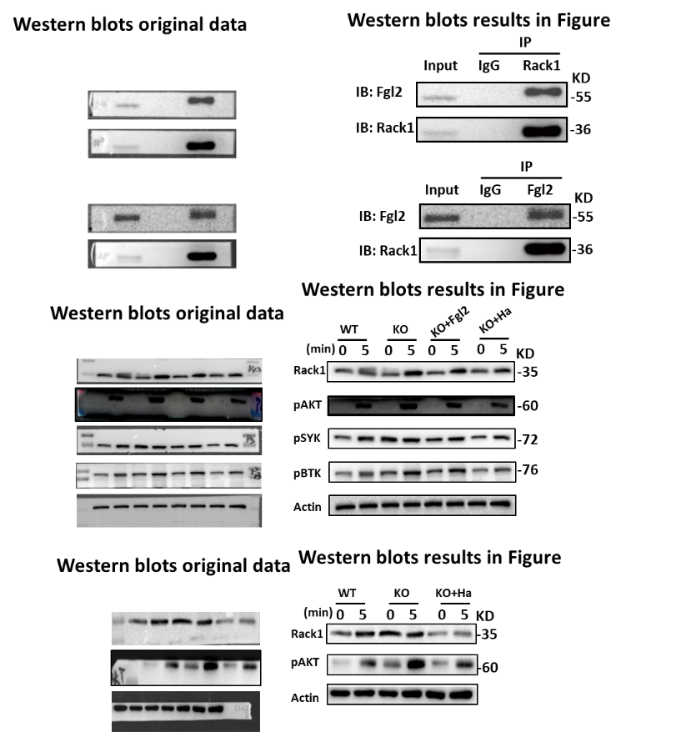


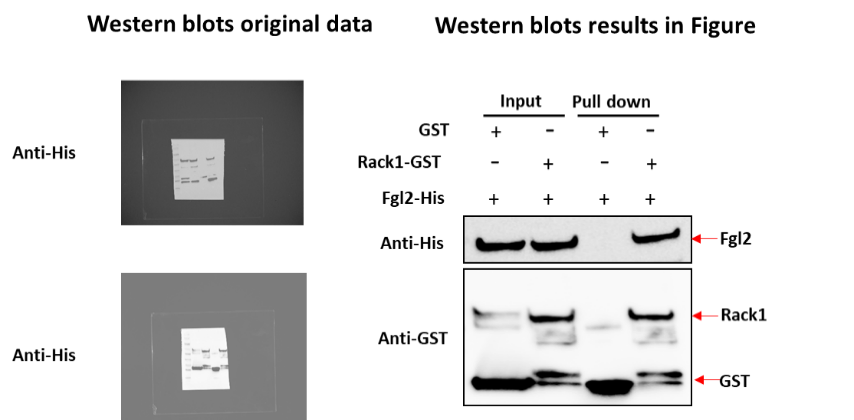

Supplement: Supplementary file 1 — Figure S1 (a) Immunofluorescence analysis was performed on the spleen sections of WT mice and WT mice infected with T.s. for Fgl2 and IgA (Fgl2 was labeled in red, and IgA in green). Representative images were acquired with a 60× objective. Scale bar, 10 µm. (b, c) Detection of Fgl2 and IgA expression levels in plasma by ELISA in WT mice and WT mice infected with T.s. (WT n = 5, KO n = 5). (d–g) Flow cytometric analysis of T follicular helper (Tfh; CD4+TCR‐β+CXCR5+PD‐1+) and regulatory B cells (Bregs; CD19+IL‐10+) in the spleen, mLNs, and PPs of T. spiralis‐infected WT and Fgl2‐KO mice (n = 5 per group). Representative plots and summary graphs of the percentages of these subsets are shown (WT n = 5, KO n = 5). (H) Quantification of Trichinella larvae recovered from the duodenum of infected WT and KO mice. Error bars represent the mean (±SD). *p < 0.05, **p < 0.01, ****p < 0.0001, ns: no significant difference. Figure S2 (a) Representative flow cytometry plots showing the percentages of IgG1+, IgG2b+, and IgE+ B cells in the spleen, mLNs, and PPs of T. spiralis‐infected WT and KO mice (n = 5 per group). (b–h) Flow cytometric analysis of splenic B cell subsets from WT and KO mice at the muscle stage of T. spiralis infection (5 weeks postinfection). Representative plots and summary graphs show the percentages of FO B cells, GC B cells, plasmablasts, plasma cells, IgA+ plasma cells, and class‐switched B cells (IgG1+, IgG2b+, IgA+, IgE+). Muscle Trichinella infection stage: After the intestinal Trichinella infection, mice were infected for more than 5 weeks, and euthanized by cervical dislocation. Spleen and all muscle tissues were collected. (j) Quantification of Trichinella larvae recovered from the muscle tissue of WT and KO mice at the muscle stage of infection (n = 5 per group). Error bars represent the mean (±SD). *p < 0.05, ***p < 0.001, ns: no significant difference. Figure S3 (a) Diagram defining genetic markers of various cell subpopulations. (b) UMAP plot show [file MCO2-7-e70633-s001.docx]
